# Supplementary material for: TORC2 inhibition triggers yeast chromosome fragmentation through misregulated Base Excision Repair of clustered oxidation events
Source: Nat Commun. 2024 Nov 15;15:9908. doi: 10.1038/s41467-024-54142-z (PMC11568337; doi:10.1038/s41467-024-54142-z)
Supplement: Supplementary file 1 — Supplementary Information [file 41467_2024_54142_MOESM1_ESM.pdf]

Supplementary Material for Shimada et al., TORC2 inhibition triggers yeast chromosome fragmentation through misregulated Base Excision Repair of clustered oxidation events

Supplementary Table 1: Yeast strains (*S. cerevisiae*) used in this study

Supplementary Table 2: Plasmids used in this study

Supplementary Fig. 1. Related imidazoquinoline-derived TORC2 inhibitors induce YCS

Supplementary Fig. 2: AP site frequency, FACS synchronization controls and modeling of DSB number based on mean fragment size with estimated B/A ratios

Supplementary Fig. 3: Degron-triggered degradation of Ogg1 and Rad1 generates N-glycosylase- and AP endonuclease-deficient strains in which YCS is restored by re-expression of *APN1*

Supplementary Fig. 4: Depletion of Pcd1 does not affect YCS

Supplementary Fig. 5: FACS controls for strains synchronized in G1 during YCS analysis

Supplementary Fig. 6: pNLS-APN1 and APN2 do not mimic TORC2 inhibition; in G1 *hnt3*Δ confers partial resistance to YCS

Supplementary Data 1: Excel sheet with quantitation of CHEF gels (also Source Data File)

Source Data Files: uncropped Western blots, mass spec analysis

Note: references in Supplementary materials refer to reference numbering in main article

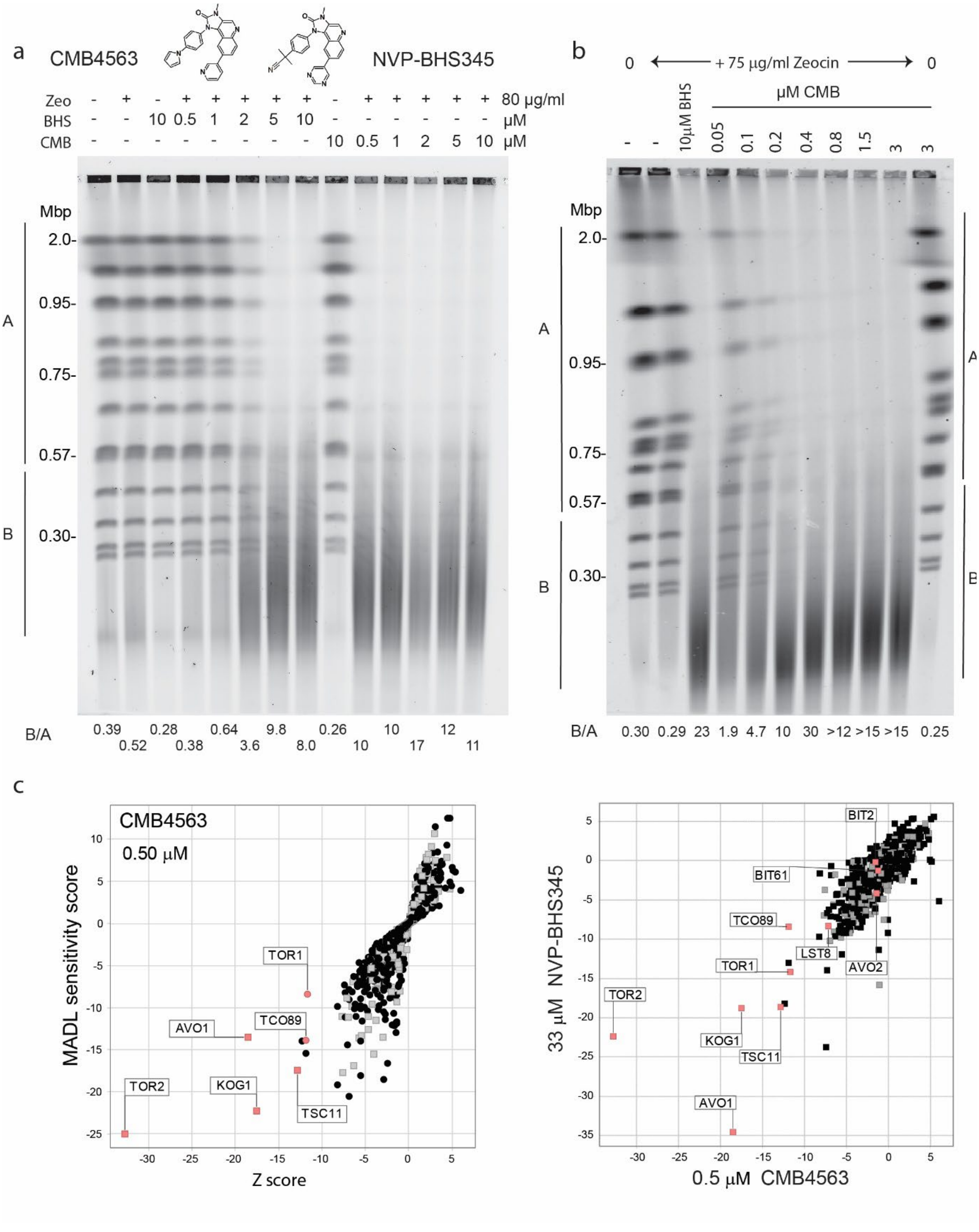

Supplementary Figure 1. Related imidazoquinoline-derived TORC2 inhibitors induce YCS

a) Exponentially cultured WT cells (GA-1981) were treated with Zeocin (80 mg/ml) and the indicated concentrations of either CMB4563 (CMB) or NVP-BHS345 (BHS) for 80 min. Genomic DNA was isolated and subjected to CHEF gel analysis and was stained with HDGreen. In the absence of Zeocin neither inhibitor triggers genomic fragmentation, and CMB elicits YCS at 10- to 20-fold lower concentrations than BHS. Quantitation of all CHEF gels shown is in Supplementary Data 1.

b) As a) exponentially growing GA-1981 cells were incubated with 75 mg/ml Zeocin and different concentrations of CMB4563 from 0.05 µM to 3µM which were compared to the effect of 10 µM NVP-BHS345. Without Zeocin, even 3µM CMB4563 did not provoke YCS.

c) Haploinsufficiency profiling (HIP) of CMB4563 (left) and comparison to HIP of NVP-BHS345 (right) according to Hoepfner et al. (ref 44). The haploinsufficiency profile of 0.5 µM of CMB suggests that it inhibits TORC2, as does BHS345 (ref. 24). Direct comparison of 33 µM BHS and 0.5 µM CMB shows strong similarity, with CMB having a stronger bias towards Tor2 kinase.

Supplementary Figure 2: AP site frequency, FACS synchronization controls and modeling of DSB number based on mean fragment size with estimated B/A ratios

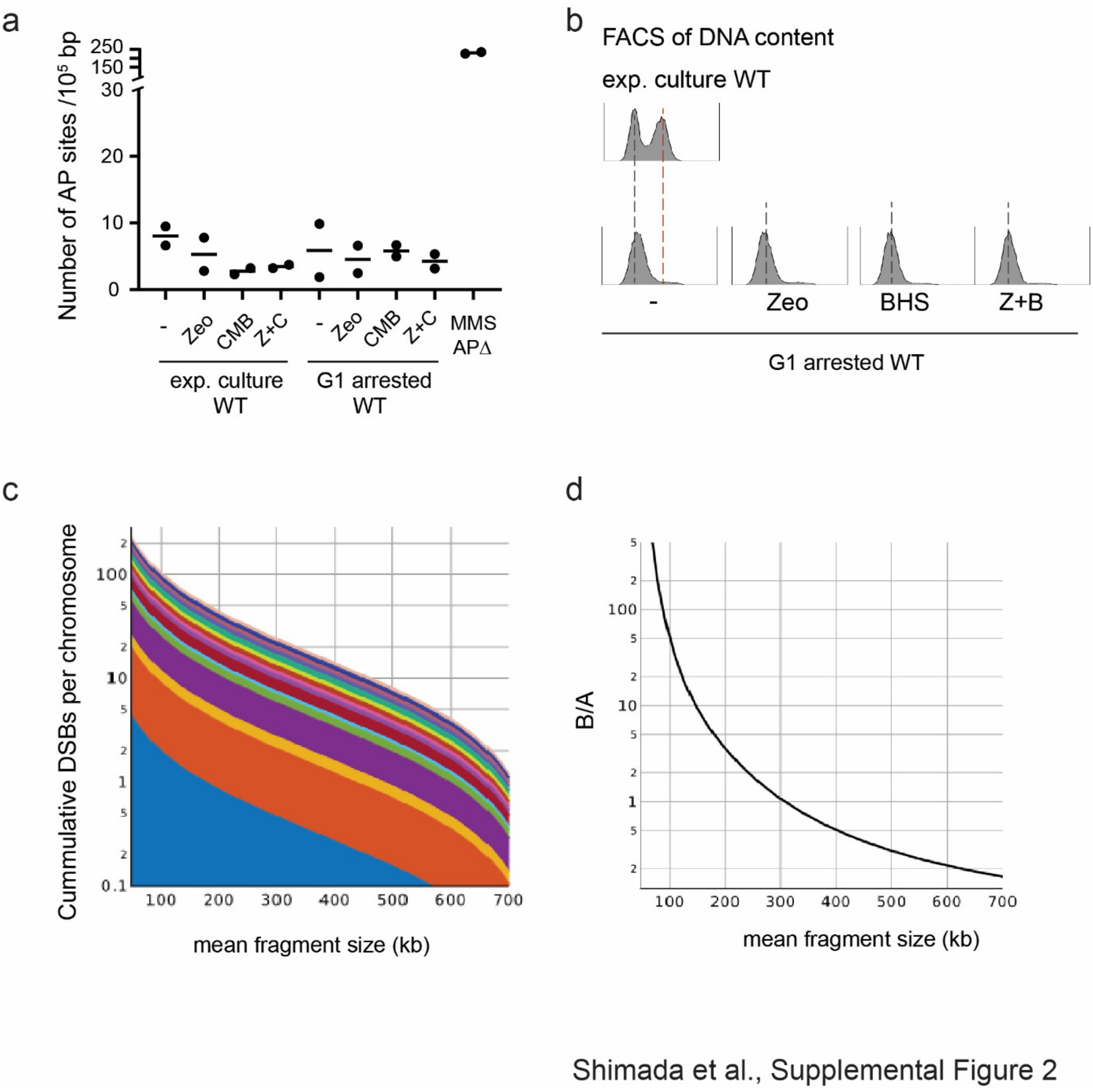

Supplementary Figure 2. AP site frequency, FACS synchronization controls and modeling of DSB number based on mean fragment size with estimated B/A ratios

a) Abasic site (AP site) quantitation. Exponentially growing wild-type (GA-8369) culture was split and half was arrested in G1 by α-factor. Both were treated with 50 μg/ml Zeocin, 0.5 μM CMB4563, or both for 90 min. In parallel, cells lacking AP endonucleases APD (GA-8509) were treated with 0.1% MMS for 90 min, after IAA treatment to deplete Rad1. AP sites were quantified with DNA damage Colorimetric Kit (BioVision) following the manufacturer's protocol on column-purified DNA (Qiagen). The plot represents an average of independent biological duplicates with the bar indicating the mean.

b) FACS analysis for G1 and exponential cultures of wild-type yeast (GA-1981) treated with BHS345 (10 mM), Zeocin (75 mg/ml), or both for 80 min were stained with propidium iodide and analyzed with FACSCalibur (Becton Dickinson) for DNA content. Vertical lines indicate 1C and 2C DNA content.

c) The distribution of fragment sizes was modeled and plotted as described in Methods. Assuming that DSBs are independently and uniformly distributed, i.e. they occur at the same frequency (λ) per unit length of DNA in all chromosomes, and can be described by a Poisson process with parameter λ, then the number of breaks in chromosome i follows a Poisson distribution of parameter λS_i, where S_i is the size of chromosome i. The mean fragment size over all the chromosomes is S_tot/(λS_tot+16), where S_tot is the length of the genome. The mean number of breaks per chromosome can be calculated as a function of the mean fragment size as follows:

1) The number of DSBs, *n,* was drawn from the Poisson distribution of parameter λS_i.

2) The locations of those *n* DSBs have the same distribution as the order statistics corresponding to *n* independent random variables uniformly distributed on the interval [0, S_i]. Hence, the locations of the *n* DSBs, $x_1, \dots, x_n$, were drawn from a uniform distribution on [0, S_i]. Those locations were then ordered such that $x_{k_1} < \dots < x_{k_n}$.

3) The fragment sizes $x_{k_1}, x_{k_2} - x_{k_1},\ x_{k_3} - x_{k_2},\ \dots,\ x_{k_n} - x_{k_{n-1}},\ S_i - x_{k_n}$ were stored.

4) To construct the distribution of fragment sizes, steps 1-3 were repeated 1000 times.

The number of breaks per chromosome as a function of average fragment size, is plotted here for values from 75 kb to 500 kb.

D) The theoretical relative intensity distribution*,* $I(x)$, of DNA fragment from randomly distributed DSBs is expressed as

$$I(x) = \lambda x e^{-\lambda x}(32 + S_{tot}\lambda - 16\lambda x)$$

(see eqn. 2 ref. 91).

The theoretical ratio B/A for B <550kb and A> 550kb, for example, is given by

$$\frac{\int_0^{5500000} I(x)dx}{\int_{550000}^{\infty} I(x)dx}$$

We calculated the B/A ratio for each value λ corresponding to a mean fragment size over all chromosomes of 70 – 700 kb. The plot of B/A vs mean fragment size is shown. Data shown results from 1000 repetitions of the modeling algorithm for randomly induced breaks.

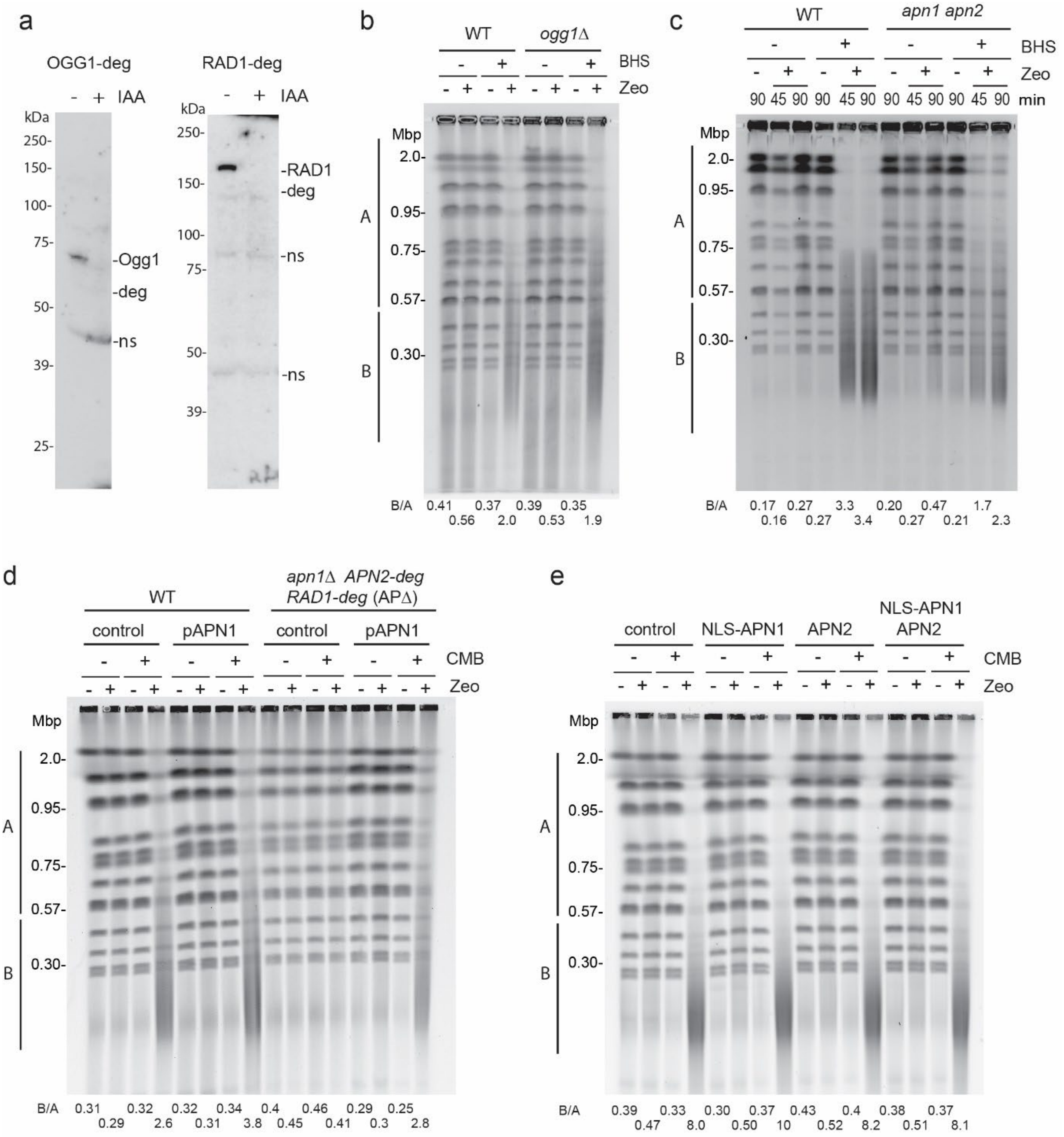

Supplementary Figure 3: Degron-triggered degradation of Ogg1 and Rad1 generates N-glycosylase- and AP endonuclease-deficient strains in which YCS is restored by re-expression of *APN1*

a) Rapid degradation of degron-tagged targets on IAA : Ogg1-3x minidegron (OGG1-deg) and AID-RAD1 (RAD1-deg) cells were exponentially cultured then treated ±0.5 mM IAA for 1h. Total protein sample was subjected to Western blot probed with anti-IAA17 antibody (gift from M. Kanemaki). The gel and Western blots are shown in their entirety.

b) The *ogg1*Δ mutant is weakly resistant to YCS. Isogenic wild-type (GA-4732) and GA-8327 (*ogg1*Δ) were incubated with the indicated reagents for 80 min prior to CHEF gel analysis

and quantitation as in Fig. 1a (see Methods). CHEF gel quantitation is in Supplementary Data 1. Other N-glycosylases complement the absence of Ogg1.

c) The *apn1*Δ *apn2*Δ mutant is very weakly resistant to YCS. Wild-type (GA-1981) and *apn1*Δ *apn2*Δ (GA-8175) cells growing exponentially in SC were treated with 75 μg/ml Zeocin, 10 μM NVP-BHS345 (BHS), or both for 45 or 90 min, prior to CHEF gel analysis and quantitation as in Fig. 1a; values are in Supplementary Data 1.

d) Isogenic wild-type (GA-8369) and *apn1 APN2-deg RAD1-deg* (GA-8509) cells were transformed by control (p415-ADH) or pAPN1 plasmid. Transformed cells were synchronized G1 were treated with 0.5 mM IAA for 1h. Cells were treated with 0.5 μM CMB4563, 50 μg/ml Zeocin, or both for 60 min, prior to CHEF gel analysis and quantitation as in Fig.1a; values are in Supplementary Data 1. pAPN1 restores YCS in AP endonuclease-deficient strains.

e) Overexpression of *APN2* and *NLS-APN1* have little impact on Zeocin-induced YCS with or without BHS. Wild-type (GA-1981) was transformed by pairs of plasmids (see Supplementary Table 2) to overexpress one or both APN endonucleases (pCM190 + pCM190HIS3, both empty vectors), *NLS-APN1* (pCM190-NLS-APN1 + pCM190HIS3), *APN2* (pCM190 + pCM190-HIS3-APN2), or *NLS-APN1* and *APN2* (pCM190-NLS-APN1 + pCM190-HIS3-APN2). Cells were grown in SC + 1 μg/ml Dox, then Dox was removed, washed twice in SC-URA-HIS minus Dox to induce expression. 0.5 μM CMB4563, 75 μg/ml Zeocin, or both were added for 80 min, prior to CHEF gel analysis and quantitation as in Fig. 1a; values are in Supplementary Data 1. Overexpressed Apn1/Apn2 do not trigger YCS on Zeocin.

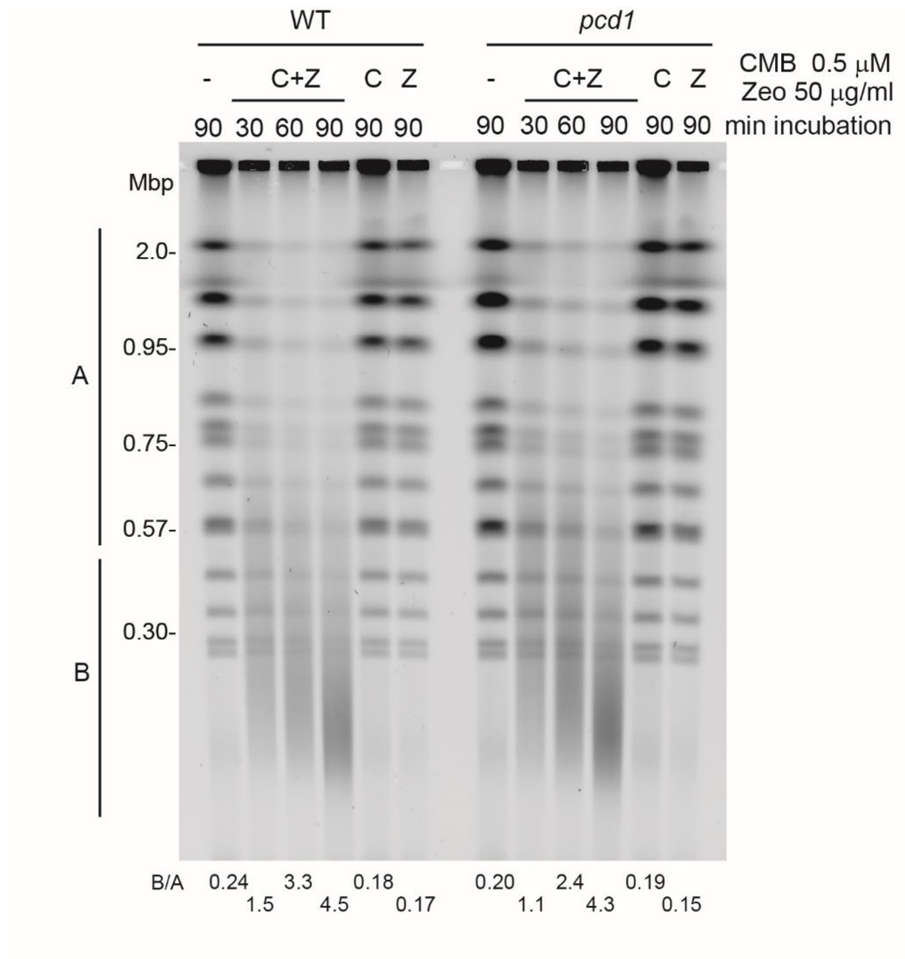

Supplementary Figure 4: **Depletion of Pcd1 does not affect YCS**

Loss of Pcd1 activity does not increase the rate of YCS. Isogenic strains GA-1981 (wild-type) and GA-10561 (*pcd1Δ*) were grown exponentially and incubated with the indicated reagents for the indicated times to induce YCS. Samples were monitored by CHEF gel analysis and quantified as in Fig. 1a. There is no significant effect of loss of Pcd1, the yeast homologue of hMTH1, which enhances the pool of free oxidized purine nucleotides. Quantitation of CHEF gel and B/A values are in Supplementary Data 1.

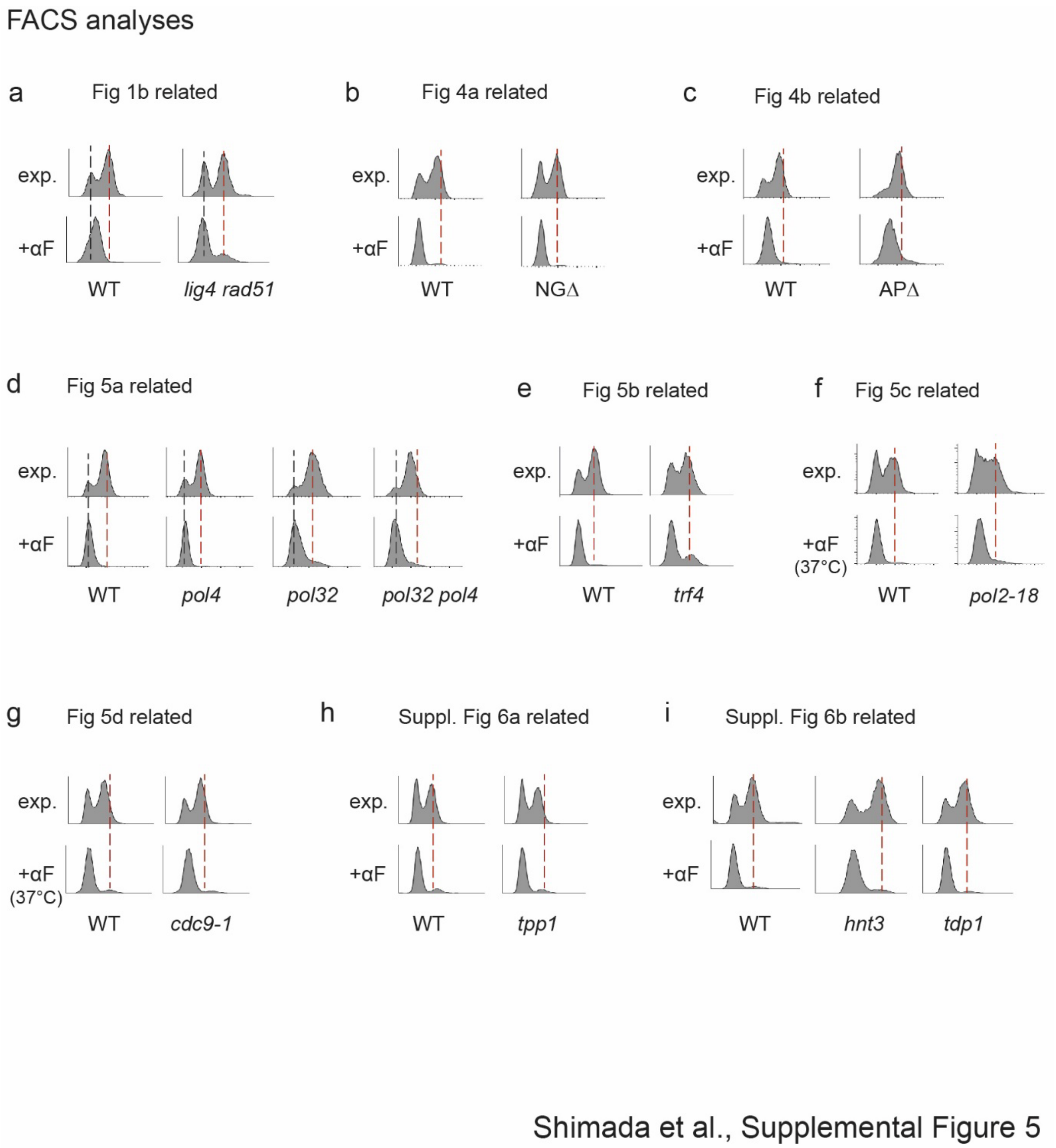

Supplementary Figure 5: FACS controls for strains synchronized in G1 during YCS analysis

FACS analysis of cells relevant to G1 arrest experiments as indicated in the figure. Cells were fixed and stained with propidium iodide and the cell cycle profile was analyzed by FACSCalibur (Becton Dickinson). All G1 arrested cells show accumulation of 1N DNA content. Dotted lines represent 1N (black) and 2N (red) content, where relevant.

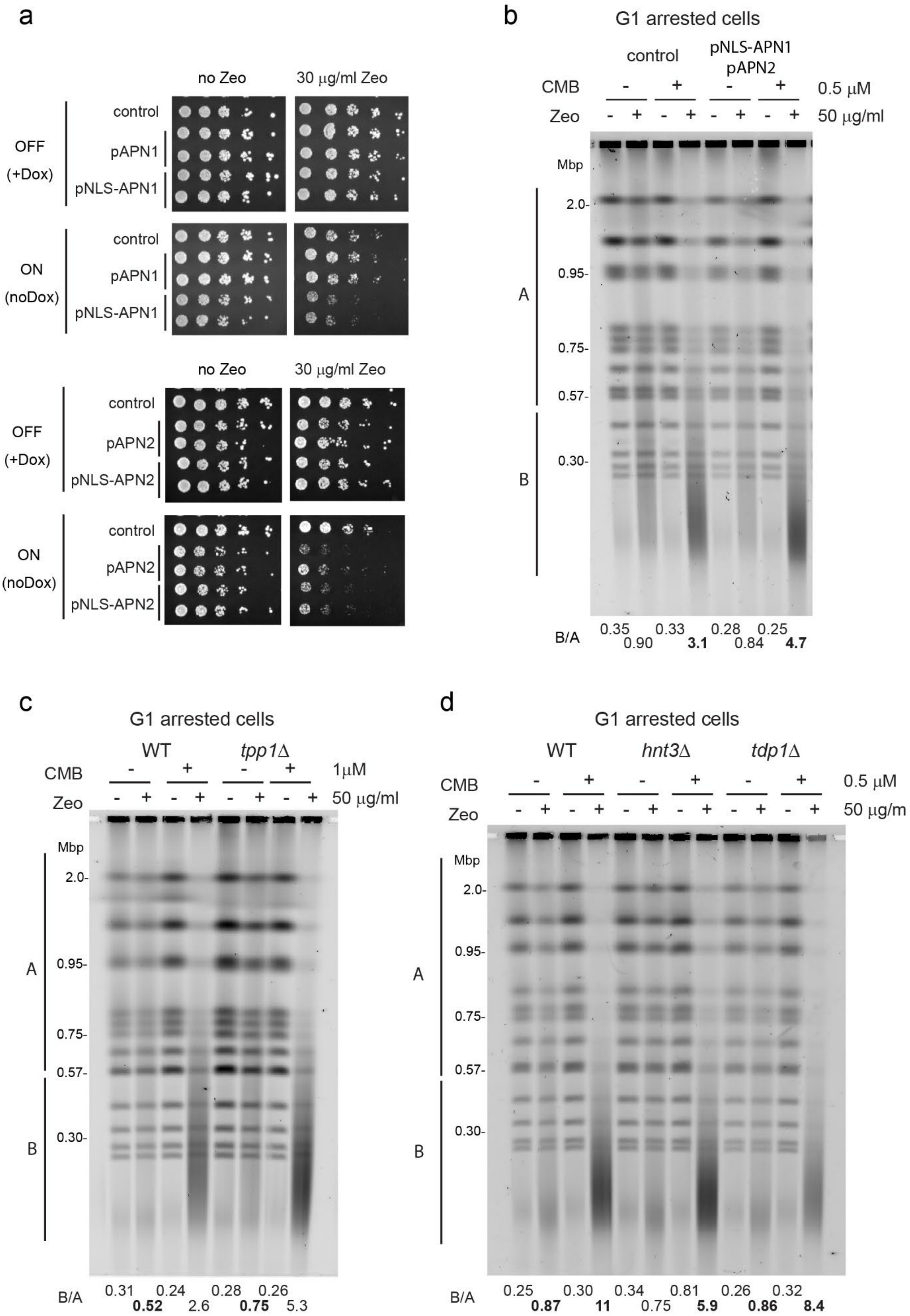
a
no Zeo
30 μg/ml Zeo
OFF
(+Dox)
ON
(noDox)
control
pAPN1
pNLS-APN1
pAPN2
pNLS-APN2
b
G1 arrested cells
control
pNLS-APN1
pAPN2
CMB
0.5 μM
Zeo
50 μg/ml
Mbp
2.0-
0.95-
0.75-
0.57-
0.30-
A
B
B/A
0.35 0.33 0.28 0.25
0.90 3.1 0.84 4.7
c
G1 arrested cells
WT
tpp1Δ
1μM
B/A
0.31 0.24 0.28 0.26
0.52 2.6 0.75 5.3
d
G1 arrested cells
WT
hnt3Δ
tdp1Δ
50 μg/m
0.25 0.30 0.34 0.81 0.26 0.32
0.87 11 0.75 5.9 0.86 8.4

Supplementary Figure 6: pNLS-APN1 and APN2 do not mimic TORC2 inhibition, and in G1 *hnt3*Δ confers partial resistance to YCS

a) Drop assay for Zeocin sensitivity upon *APN1* or *APN2* overexpression. Wild-type GA-1981 cells were transformed with plasmids expressing *APN1* (pAPN1), nuclear localization signal (NLS) fused *APN1* (pNLS-APN1), *APN2* (pAPN2), NLS-fused *APN2* (pNLS-APN2), or pCM190 (no insert) all under the same Tet-off promoter (ref. 97). Cells growing exponentially in SC-uracil (URA) plus 2 μg/ml doxycycline (Dox), were washed once in SC-URA minus Dox, and 5-fold serially diluted and spotted on SC-URA ±1 μg/ml Dox and ±30 μg/ml Zeocin, as indicated. Images were taken after 3 days incubation at 30°C. Cells were plated in triplicate, and experiment was repeated twice.

b) Cells carrying the control or Tet-off (ref. 97) pNLS-APN1 and pAPN2 plasmids were cultured with 2 μg/ml Dox. Dox was removed by washing, and cells were cultured without Dox for 2 hours, and arrested in G1 with α-factor. Cells were treated with indicated reagents for 60 min and genomic DNA was subjected to CHEF gel for YCS analysis. Excess Apn1/Apn2 enhances YCS but does not mimic TORC2 inhibition on Zeocin. Quantitation of CHEF gels and B/A are in Supplementary Data 1.

c) Isogenic strains of wild-type GA-4732 and *tpp1Δ* GA-10894 in exponentially growth were arrested in G1 by α-factor for 1.5h. Cells were incubated with the indicated reagents for 100 min. Genomic DNA was isolated and analysis by CHEF gel and quantified as in Fig. 1a. FACS is in Supplementary Fig. 5. Quantitation of CHEF gels and B/A are in Supplementary Data 1.

d) Loss of Hnt3 slightly reduces YCS while loss of Tdp1 does not in G1-arrested cells. Isogenic strains wildtype (GA-1981) and *hnt3Δ* (GA-10565) or *tdp1Δ* (GA-10597) cell growing exponentially were arrested in G1 by α-factor for 1.5h. YCS assay was performed as in panel c, except that incubation was for 80 min. Values show that in the absence of CMB, the *tdp1*Δ does not trigger significant breakage, unlike *tpp1*Δ, which shows an increase (panel c). Whereas *hnt3Δ* has little effect in the absence of CMB, on CMB and Zeocin YCS efficiency drops. FACS is in Supplementary Fig. 5. Quantitation of CHEF gels and B/A are in Supplementary Data 1.

Supplementary Table 1. Yeast strains (*S. cerevisiae*) used in this study.

Name	Genotype	Source
**GA-741**	MATa *ade5-1 leu2-3,112 trp1-289 pol2-3::LEU2 yCpPOL2*	Ref 96 (Araki et al., 1992)
**GA-742**	MATa *ade5-1 leu2-3,112 trp1-289 pol2-3::LEU2 yCppol2-18*	Ref 96 (Araki et al., 1992)
**GA-1981**	MATa *ade2-1 trp1-1 his3-11,15 ura3-1 leu2-3,112 can1-100 RAD5*	H. Klein(HKY579-10A)
**GA-4732**	MATa *his3Δ1 leu2Δ0 met15Δ0 ura3Δ0*	K. Shirahige (BY4741)
**GA-6908**	GA-1981 with *lig4::HIS3 rad51::TRP1*	Ref. 24 Shimada et al., 2013
**GA-8175**	GA-1981 with *apn1::KanMX4 apn2::CaURA3*	This study
**GA-8327**	GA-4732 with *ogg1::KanMX4*	OpenBiosystems
**GA-8369**	GA-1981 with *ADH1_OsTIR1::URA3*	This study
**GA-8457**	GA-8369 with *OGG1-3x mini-degron_HIS3MX ntg1::KanMX4 ntg2::natMX4*	This study
**GA-8509**	GA-8369 with *AID-RAD1_KanMX apn1::KanMX4 APN2-3x mini-degron_HIS3MX*	This study
**GA-8555**	GA-1981 with *OGG1-9Myc_natNT2*	This study
**GA-8692**	GA-1981 with *APN1-9Myc_natNT2*	This study
**GA-8708**	GA-8709 with *cdc9-1*	This study
**GA-8709**	MATa *ade2-1 his3-11,15 ura3-1*	This study
**GA-9683**	GA-1981 with *rev3::KanMX4*	This study
**GA-9686**	GA-1981 with *pol32::natMX4*	This study
**GA-10504**	GA-1981 with *APN1-yeGFP_hphNT1 NUP49-RFP_caURA3*	This study
**GA-10561**	GA-1981 with *pcd1::CaURA3*	This study
**GA-10565**	GA-1981 with *hnt3::CaURA3*	This study
**GA-10595**	GA-1981 with *pol4::hphMX4*	This study
**GA-10597**	GA-1981 with *tdp1::hphMX4*	This study
**GA-10607**	GA-1981 with *pol4::hphMX4 pol32::natMX4*	This study
**GA-10632**	GA-1981 with *trf4::KanMX6*	This study
**GA-10894**	GA-4732 with *tpp1::KanMX4*	OpenBiosystems
**GA-10997**	GA1981 with *pol3-ct* (*pol3-ct-1* in Fig.3)	L. Maloisel (L96.4C)
**GA-11000**	GA1981 with *pol3-ct* (*pol3-ct-2* in Fig.3)	S. Gangloff (LM118-1A)

Supplementary Table 2. Plasmids used in this study

Internal No	name	description	Source
#732	p415-ADH	CEN/ARS, *ADH1* promoter, *LEU2*, AmpR	Ref 90, Mumberg et al. 1995
#975	p415-GAL1	CEN/ARS, GAL1 promoter, *LEU2*, AmpR	Ref 89, Mumberg et al. 1994
#878	pCM190	2μ, URA3, tet-off promoter, tTA, AmpR	Ref 97, Gari et al., 1997
#2957	pCM190-ACT1	#878 with *ACT1*	This study
#3550	p415-APN1	#732 with *APN1*	This study
#3356	pCM190-act1-nes	#878 with act1-nes	This study
#3358	pCM190-act1-S14C-nes	#878 with *act1-S14C*-nes	This study
#3467	pCM190-AP-act1-nes	#878 with *AP-act1-nes*	This study
#3861	pCM190-act1-111-nes	#878 with *act1-111-nes*	This study
#3952	pCM190-APN1	#878 with *APN1*	This study
#3955	pCM190-NLS-APN1	#878 with *NLS-APN1*	This study
#3957	pCM190-HIS3	#878 *URA3* swapped to *HIS3*	This study
#3959	pCM190-APN2	#878 with *APN2*	This study
#3960	pCM190-NLS-APN2	#878 with *NLS-APN2*	This study
#3965	pCM190-HIS3-APN2	#3957 with *APN2*	This study
#4046	p415-GAL1-Dam	#975 with Dam-methylase	This study

Supplementary Data 1. Quantitation of CHEF gels (Excel sheet)

All CHEF gels shown (and repetitions) were quantified as described in Methods and values are provided in the Excel sheet along with B/A ratios. The quantitation was performed on Typhoon generated digital images which had linearity over a range of 10^4 units, and intensity values including B/A ratios were determined in a blinded fashion.
