# Supplementary material for: TORC2 inhibition triggers yeast chromosome fragmentation through misregulated Base Excision Repair of clustered oxidation events
Source: Nat Commun. 2024 Nov 15;15:9908. doi: 10.1038/s41467-024-54142-z (PMC11568337; doi:10.1038/s41467-024-54142-z)
Supplement: Supplementary file 2 — Description of Additional Supplementary Files [file 41467_2024_54142_MOESM2_ESM.pdf]

Description of Additional Supplementary Files

File Name: Supplementary Data 1
Description: **Quantitation of CHEF gels (Excel sheet)**
All CHEF gels shown (and repetitions) were quantified as described in Methods, and the intensity values are provided in this Excel sheet along with B/A ratios. The quantitation was performed on Typhoon generated digital images which had linearity over a range of 10^4 units; intensity values and B/A ratios were determined in a blinded fashion.
