# Supplementary material for: TORC2 inhibition triggers yeast chromosome fragmentation through misregulated Base Excision Repair of clustered oxidation events
Source: Nat Commun. 2024 Nov 15;15:9908. doi: 10.1038/s41467-024-54142-z (PMC11568337; doi:10.1038/s41467-024-54142-z)
Supplement: Supplementary file 4 — Reporting Summary [file 41467_2024_54142_MOESM4_ESM.pdf]

nature portfolio

Corresponding author(s): Susan M Gasser

Last updated by author(s): Sep 30, 2024

Reporting Summary

Nature Portfolio wishes to improve the reproducibility of the work that we publish. This form provides structure for consistency and transparency in reporting. For further information on Nature Portfolio policies, see our Editorial Policies and the Editorial Policy Checklist.

Statistics

For all statistical analyses, confirm that the following items are present in the figure legend, table legend, main text, or Methods section.

n/a	Confirmed	
☐	☒	The exact sample size (*n*) for each experimental group/condition, given as a discrete number and unit of measurement
☐	☒	A statement on whether measurements were taken from distinct samples or whether the same sample was measured repeatedly
☐	☒	The statistical test(s) used AND whether they are one- or two-sided<br>*Only common tests should be described solely by name; describe more complex techniques in the Methods section.*
☒	☐	A description of all covariates tested
☐	☒	A description of any assumptions or corrections, such as tests of normality and adjustment for multiple comparisons
☐	☒	A full description of the statistical parameters including central tendency (e.g. means) or other basic estimates (e.g. regression coefficient) AND variation (e.g. standard deviation) or associated estimates of uncertainty (e.g. confidence intervals)
☐	☒	For null hypothesis testing, the test statistic (e.g. *F*, *t*, *r*) with confidence intervals, effect sizes, degrees of freedom and *P* value noted<br>*Give P values as exact values whenever suitable.*
☒	☐	For Bayesian analysis, information on the choice of priors and Markov chain Monte Carlo settings
☒	☐	For hierarchical and complex designs, identification of the appropriate level for tests and full reporting of outcomes
☒	☐	Estimates of effect sizes (e.g. Cohen's *d*, Pearson's *r*), indicating how they were calculated

Our web collection on statistics for biologists contains articles on many of the points above.

Software and code

Policy information about availability of computer code

Data collection	Gel scans are acquired with commercially available software and tools (Typhoon) are described in Methods
Data analysis	Gel scan values were analysed as described in the Methods

For manuscripts utilizing custom algorithms or software that are central to the research but not yet described in published literature, software must be made available to editors and reviewers. We strongly encourage code deposition in a community repository (e.g. GitHub). See the Nature Portfolio guidelines for submitting code & software for further information.

Data

Policy information about availability of data

All manuscripts must include a data availability statement. This statement should provide the following information, where applicable:

- Accession codes, unique identifiers, or web links for publicly available datasets
- A description of any restrictions on data availability
- For clinical datasets or third party data, please ensure that the statement adheres to our policy

all data is included in the submission either in figures or as excel files

Research involving human participants, their data, or biological material

Policy information about studies with human participants or human data. See also policy information about sex, gender (identity/presentation), and sexual orientation and race, ethnicity and racism.

Reporting on sex and gender	na
Reporting on race, ethnicity, or other socially relevant groupings	na
Population characteristics	na
Recruitment	na
Ethics oversight	na

Note that full information on the approval of the study protocol must also be provided in the manuscript.

Field-specific reporting

Please select the one below that is the best fit for your research. If you are not sure, read the appropriate sections before making your selection.

☒ Life sciences ☐ Behavioural & social sciences ☐ Ecological, evolutionary & environmental sciences

For a reference copy of the document with all sections, see nature.com/documents/nr-reporting-summary-flat.pdf

Life sciences study design

All studies must disclose on these points even when the disclosure is negative.

Sample size	Where relevant, number of cells counted or used in an experiment is included
Data exclusions	none
Replication	indicated in legends and supplemental material
Randomization	not relevant except for the modeling of frequency of breaks and this is mathematical randomization
Blinding	Where relevant, double blind approaches were used for quantifying images

Reporting for specific materials, systems and methods

We require information from authors about some types of materials, experimental systems and methods used in many studies. Here, indicate whether each material, system or method listed is relevant to your study. If you are not sure if a list item applies to your research, read the appropriate section before selecting a response.

Materials & experimental systems

n/a	Involved in the study	
☐	☒	Antibodies
☒	☐	Eukaryotic cell lines
☒	☐	Palaeontology and archaeology
☒	☐	Animals and other organisms
☒	☐	Clinical data
☒	☐	Dual use research of concern
☒	☐	Plants

Methods

n/a	Involved in the study	
☒	☐	ChIP-seq
☐	☒	Flow cytometry
☒	☐	MRI-based neuroimaging

Antibodies

Antibodies used	anti-actin (Millipore, Mab1501 clone C4) used 1:4000, anti-MCM2 (Santa Cruz, SC-6680) used 1:2000 dilution, rat anti-tubulin (YOL 1/34, ab6161, Abcam) used 1:10000 dilution, anti-tubulin (Thermo Fisher Scientific: MA1-80017), and anti-MYC (9E10) prepared in house, used 1:100 dilution, rabbit anti-histone H3 (polyclonal, ab1791, Abcam) used 1:1000 dilution, rabbit anti-histone H4 (polyclonal, ab10158, Abcam) used 1:5000 dilution, rabbit anti-Orc2 (Gasser laboratory, validated in93 used 1:1000 dilution, and anti-

	Rad53 (mouse monoclonal; 11G3G6 hybridoma clone, Gasser laboratory, used 1:200. Rabbit anti-Arp5 polyclonal was a kind gift of Dr M. Harata (Tohoku University, Japan), validated use at 1:10000 dilution; rabbit anti-IAA17 was a kind gift of Dr M. Kanemaki (NIG, Japan) used 1:1000 dilution. mouse anti-fluorescein (FITC) antibody (Jackson Immuno Res., clone IF8-IE4)
Validation	negative controls are used to confirm specificity of reagents and references were cited when done elsewhere

Plants

Seed stocks	na
Novel plant genotypes	na
Authentication	na

Flow Cytometry

Plots

Confirm that:

☒ The axis labels state the marker and fluorochrome used (e.g. CD4-FITC).

☒ The axis scales are clearly visible. Include numbers along axes only for bottom left plot of group (a 'group' is an analysis of identical markers).

☒ All plots are contour plots with outliers or pseudocolor plots.

☒ A numerical value for number of cells or percentage (with statistics) is provided.

Methodology

Sample preparation	FACS was used to determine DNA content in a population of yeast cells that were fixed and stained with propidium iodide
Instrument	Cell cycle profile (1N or 2N DNA) was analyzed by FACSCalibur (Becton Dickinson).
Software	The raw trace is shown, there was no unique software used
Cell population abundance	The DNA content of cells was determined as a trace with peaks at 1N and 2N
Gating strategy	No specific gating strategy was used, because the cells were not SORTED but only monitored

☒ Tick this box to confirm that a figure exemplifying the gating strategy is provided in the Supplementary Information.
