# Supplementary material for: TORC2 inhibition triggers yeast chromosome fragmentation through misregulated Base Excision Repair of clustered oxidation events
Source: Nat Commun. 2024 Nov 15;15:9908. doi: 10.1038/s41467-024-54142-z (PMC11568337; doi:10.1038/s41467-024-54142-z)
Supplement: Supplementary file 6 — Source Data [file 41467_2024_54142_MOESM6_ESM.zip › raw data Fig 7a 160330_Act_bicycl_peptide_PD.pdf]

User: Kenji Shimada (shimada)
Group: Gasser, S.
Operator: Ragna Sack

Date of submission: 30.03.2016
Date of report: 04.04.2016

Project Name : actin bicyclic peptide pull down
Project ID : 476
Experiment Name : 160330_pull down
Experiment ID : 1305

Experimental Information

Experiment Description	:	I have performed pull-down experiment with bicyclic peptide which recognizes F- or F/G-actin (peRG24; F-, peRG6; F/G-, TATA2 (control)). We found that the actin was efficiently pulled out by both peptides. We also saw several common bands in both peptide pull down, and interestingly two specific bands only from F/G-actin probe. We wish to identify those actin associated factors.
Type of Analysis	:	Identification
Sample Type	:	gel
Analysis Details	:	peRG24; F-, peRG6; F/G-, TATA2 (control) pull down was performed. Protein samples were treated with IAA then subjected to SDS-PAGE. The gel was stained with commassie. The factors of our interest were numbered in the image below. (Note: This is an image of a silver stained gel)
Sample Amount	:	unknown
Number of Samples	:	15
Species 1	:	Baker's Yeast
Cys Modification	:	Iodoacetamide (IAA) before gel -> Carbamidomethyl (C)
Sample is	:	digested
Enzyme(s) for digestion	:	Trypsin

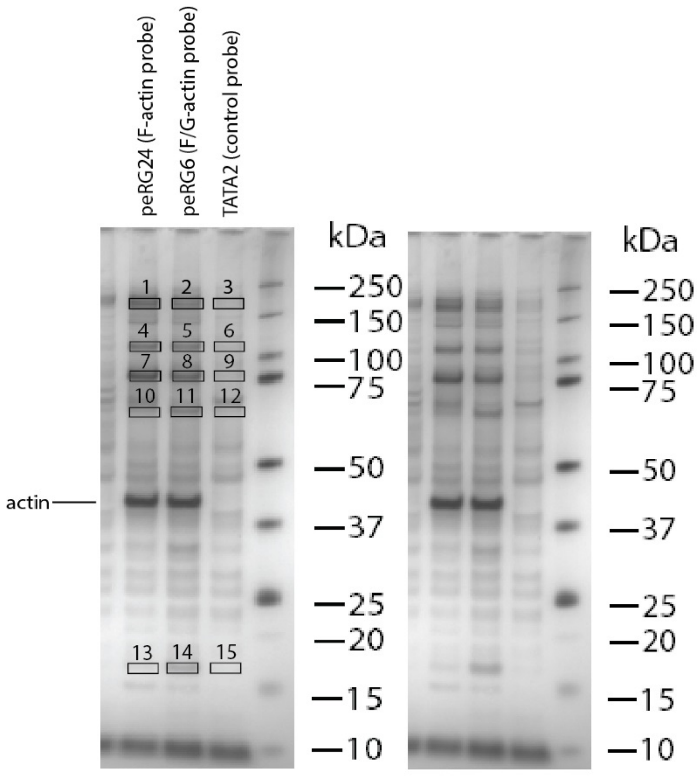

silver staining

Results

Analyses were evaluated with Scaffold. In the tables below values shown are Exclusive spectral count for protein and peptide probabilities set to 805 and two peptides per protein were required.

Remark: It can be helpful to switch to Quantitative values if the difference in number of spectra is small.

1. samples 13, 14 15

Probability Legend:
- over 95%
- 80% to 94%
- 50% to 79%
- 20% to 49%
- 0% to 19%

MS/MS View: Identified Proteins (66/81)

#	Visible?	Starred?	Identified Proteins (66/81)	Accession Number	Molecular Weight	Protein Grouping Ambiguity	17 kDa s13 (F081523)	s14 (F081535)	s15 (F081511)
1	☑	★	COF1 SGDID:S000003973, Chr XII from 40221-39804,40414-40...	YLL050C	16 kDa		36	123	1
2	☑	★	SNC2 SGDID:S000005854, Chr XV from 931081-930734, Genom...	YOR327C	13 kDa	★	9	17	5
3	☑	★	HHF1 SGDID:S000000213, Chr II from 255684-255373, Genom...	YBR009C (+1)	11 kDa		4	6	1
4	☑	★	ACT1 SGDID:S000001855, Chr VI from 54377-53260,54696-54...	YFL039C	42 kDa	★	6	8	
5	☑	★	CRN1 SGDID:S000004421, Chr XII from 990777-992732, Geno...	YLR429W	73 kDa		3	4	0
6	☑	★	NHP6B SGDID:S000002157, Chr II from 426489-426190, Geno...	YBR089C-A	11 kDa		3	5	1
7	☑	★	RFA3 SGDID:S000003709, Chr X from 96529-96161, Genome R...	YJL173C	14 kDa		0	9	
8	☑	★	MLC1 SGDID:S000003074, Chr VII from 306560-307009, Geno...	YGL106W	16 kDa		5	0	
9	☑	★	NCB2 SGDID:S000002805, Chr IV from 1266769-1266366,1266...	YDR397C	17 kDa		1	2	
10	☑	★	RPL38 SGDID:S000004317, Chr XII from 781379-781143, Geno...	YLR325C	9 kDa		1	3	
11	☑	★	UBC5 SGDID:S000002466, Chr IV from 569633-569234,569770...	YDR059C	16 kDa		0	3	
12	☑	★	YEF3 SGDID:S000004239, Chr XII from 636780-639914, Geno...	YLR249W	116 kDa		0	2	0
13	☑	★	CMD1 SGDID:S000000313, Chr II from 458362-457919, Genom...	YBR109C	16 kDa		2		
14	☑	☆	RPL26B SGDID:S000003266, Chr VII from 555812-555830,556...	YGR034W	14 kDa	★	48	53	50
15	☑	☆	RPL35B SGDID:S000002295, Chr IV from 217600-217602,2180...	YDL136W (+1)	14 kDa		23	37	34
16	☑	☆	RPS14B SGDID:S000003727, Chr X from 73787-73796,74205-7...	YJL191W	15 kDa		29	37	25

For whole list see file 160330_Act_bc_pep_PD_13_14_15.sf3.

2. samples 10, 11, 12

Probability Legend:
- over 95%
- 80% to 94%
- 50% to 79%
- 20% to 49%
- 0% to 19%

MS/MS View: Identified Proteins (136/147)

#	Visible?	Starred?	Identified Proteins (136/147)	Accession Number	Molecular Weight	Protein Grouping Ambiguity	65 kDa s10 (F081524)	s11 (F081536)	s12 (F081514)
1	☑	★	SSA2 SGDID:S000003947, Chr XII from 97485-...	YLL024C	69 kDa	★	41	44	2
2	☑	★	RPL40A SGDID:S000001410, Chr IX from 68708...	YIL148W (+3)	15 kDa		24	27	2
3	☑	★	SAC6 SGDID:S000002536, Chr IV from 715247-...	YDR129C	72 kDa		39	49	2
4	☑	★	MRN1 SGDID:S000006105, Chr XVI from 19778...	YPL184C	69 kDa		5	18	2
5	☑	★	RFA1 SGDID:S000000065, Chr I from 158619-1...	YAR007C	70 kDa		28	225	14
6	☑	★	PDI1 SGDID:S000000548, Chr III from 50221-4...	YCL043C	58 kDa		52	0	3
7	☑	★	ACT1 SGDID:S000001855, Chr VI from 54377-5...	YFL039C	42 kDa	★	9	16	0
8	☑	★	KAR2 SGDID:S000003571, Chr X from 381327-3...	YJL034W	74 kDa	★	10	13	0
9	☑	★	KRS1 SGDID:S000002444, Chr IV from 525440-...	YDR037W	68 kDa		8	12	1
10	☑	★	ILV2 SGDID:S000004714, Chr XIII from 484084...	YMR108W	75 kDa		8	15	1
11	☑	★	ECM25 SGDID:S000003737, Chr X from 54379-5...	YJL201W	68 kDa		1	10	1
12	☑	★	MAE1 SGDID:S000001512, Chr XI from 384725-...	YKL029C	74 kDa		5	14	1
13	☑	★	SRP68 SGDID:S000006164, Chr XVI from 88517...	YPL243W	69 kDa		4	14	2
14	☑	★	GGA2 SGDID:S000001150, Chr VIII from 32830...	YHR108W	64 kDa		10	16	4
15	☑	★	SRV2 SGDID:S000005082, Chr XIV from 366741...	YNL138W	58 kDa		10	34	5
16	☑	★	CBP2 SGDID:S000001030, Chr VIII from 25509-...	YHL038C	74 kDa		1	12	5
17	☑	★	RRB1 SGDID:S000004738, Chr XIII from 53469...	YMR131C	57 kDa		6	14	5
18	☑	☆	FUI1 SGDID:S000000138, Chr II from 140260-1...	YBL042C	72 kDa		0	2	
19	☑	☆	SNF1 SGDID:S000002885, Chr IV from 1412373...	YDR477W	72 kDa			2	
20	☑	☆	COF1 SGDID:S000003973, Chr XII from 40221-3...	YLL050C	16 kDa		2	10	

For whole list see file 160330_Act_bc_pep_PD_10_11_12.sf3

3. samples 7, 8, 9

Probability Legend: over 95% · 80% to 94% · 50% to 79% · 20% to 49% · 0% to 19%

#	Visible?	Starred?	MS/MS View: Identified Proteins (113/124)	Accession Number	Molecular Weight	Protein Grouping Ambiguity	s07 (F081525)	s08 (F081537)	s09 (F081516)
								80 kDa	
1	☑	★	CRN1 SGDID:S000004421, Chr XII from 990...	YLR429W	73 kDa		595	450	14
2	☑	★	PMA1 SGDID:S000002976, Chr VII from 482...	YGL008C	100 kDa	★	125	123	53
3	☑	★	NSP1 SGDID:S000003577, Chr X from 36578...	YJL041W	87 kDa		38	47	17
4	☑	★	LAS17 SGDID:S000005707, Chr XV from 675...	YOR181W	68 kDa		25	39	2
5	☑	★	MKT1 SGDID:S000005029, Chr XIV from 467...	YNL085W	94 kDa		16	39	4
6	☑	★	PRT1 SGDID:S000005888, Chr XV from 1017...	YOR361C	88 kDa		17	29	4
7	☑	★	SSE1 SGDID:S000006027, Chr XVI from 352...	YPL106C	77 kDa	★	16	22	6
8	☑	★	RPL40A SGDID:S000001410, Chr IX from 68...	YIL148W (+3)	15 kDa		13	15	0
9	☑	★	RFA1 SGDID:S000000065, Chr I from 15861...	YAR007C	70 kDa			27	
10	☑	★	PDI1 SGDID:S000000548, Chr III from 5022...	YCL043C	58 kDa		25		0
11	☑	★	HSC82 SGDID:S000004798, Chr XIII from 63...	YMR186W	81 kDa	★	60	80	54
12	☑	★	UTP4 SGDID:S000002732, Chr IV from 1116...	YDR324C	88 kDa		12	20	6
13	☑	★	SEC27 SGDID:S000003105, Chr VII from 249...	YGL137W	99 kDa		10	16	4
14	☑	★	YPP1 SGDID:S000003430, Chr VII from 8946...	YGR198W	95 kDa		12	14	2
15	☑	★	ACT1 SGDID:S000001855, Chr VI from 5437...	YFL039C	42 kDa		9	13	
16	☑	★	SAS10 SGDID:S000002312, Chr IV from 183...	YDL153C	70 kDa		10	11	3
17	☑	★	VRP1 SGDID:S000004329, Chr XII from 805...	YLR337C	83 kDa		4	13	
18	☑	★	TAF5 SGDID:S000000402, Chr II from 61852...	YBR198C	89 kDa		3	11	0
19	☑	★	SLA2 SGDID:S000005187, Chr XIV from 188...	YNL243W	109 kDa		5	3	
20	☑	★	YNL247W SGDID:S000005191, Chr XIV from ...	YNL247W	88 kDa		3	2	0
21	☑	★	RSN1 SGDID:S000004879, Chr XIII from 798...	YMR266W	108 kDa		3	4	0
22	☑	☆	VPS1 SGDID:S000001709, Chr XI from 4427...	YKR001C	79 kDa		12	35	70
23	☑	☆	GUS1 SGDID:S000003214, Chr VII from 390...	YGL245W	81 kDa		17	30	26
24	☑	☆	THS1 SGDID:S000001340, Chr IX from 2124...	YIL078W	85 kDa		21	17	12
25	☑	☆	PBP1 SGDID:S000003410, Chr VII from 8532...	YGR178C	79 kDa		11	27	19

For whole list see file 160330_Act_bc_pep_PD_07_08_09.sf3

4. samples 4, 5, 6

Probability Legend: over 95% · 80% to 94% · 50% to 79% · 20% to 49% · 0% to 19%

#	Visible?	Starred?	MS/MS View: Identified Proteins (98/109)	Accession Number	Molecular Weight	Protein Grouping Ambiguity	s04 (F081530)	s05 (F081538)	s06 (F081517)
								120 kDa	
1	☑	★	CRN1 SGDID:S000004421, Chr XII from 990...	YLR429W	73 kDa		62	40	0
2	☑	★	VRP1 SGDID:S000004329, Chr XII from 805...	YLR337C	83 kDa		34	68	1
3	☑	★	SLA2 SGDID:S000005187, Chr XIV from 188...	YNL243W	109 kDa		137	129	18
4	☑	★	PFK2 SGDID:S000004818, Chr XIII from 674...	YMR205C	105 kDa	★	93	80	42
5	☑	★	PFK1 SGDID:S000003472, Chr VII from 9737...	YGR240C	108 kDa	★	113	115	55
6	☑	★	URB2 SGDID:S000003802, Chr X from 51376...	YJR041C	135 kDa			20	0
7	☑	★	YMR124W SGDID:S000004731, Chr XIII fro...	YMR124W	106 kDa			28	0
8	☑	★	TIF4632 SGDID:S000003017, Chr VII from 4...	YGL049C	104 kDa		0	12	0
9	☑	★	RPG1 SGDID:S000000283, Chr II from 3982...	YBR079C	110 kDa		0	59	1
10	☑	★	MSH2 SGDID:S000005450, Chr XV from 147...	YOL090W	109 kDa		3	25	4
11	☑	★	YEF3 SGDID:S000004239, Chr XII from 6367...	YLR249W	116 kDa		4	50	12
12	☑	★	SEC26 SGDID:S000002646, Chr IV from 940...	YDR238C	109 kDa		10	5	21
13	☑	★	MSH3 SGDID:S000000688, Chr III from 279...	YCR092C	117 kDa		0	16	
14	☑	★	RPL40A SGDID:S000001410, Chr IX from 68...	YIL148W (+3)	15 kDa		7	10	
15	☑	★	COP1 SGDID:S000002304, Chr IV from 1981...	YDL145C	136 kDa			15	0
16	☑	★	ACT1 SGDID:S000001855, Chr VI from 5437...	YFL039C	42 kDa		5	10	0
17	☑	★	ARP8 SGDID:S000005667, Chr XV from 5925...	YOR141C	100 kDa		10	4	1
18	☑	★	RSN1 SGDID:S000004879, Chr XIII from 798...	YMR266W	108 kDa		14	13	2
19	☑	★	ENA1 SGDID:S000002447, Chr IV from 5384...	YDR040C	120 kDa	★	8	11	2
20	☑	★	PMA1 SGDID:S000002976, Chr VII from 482...	YGL008C	100 kDa		11	10	3
21	☑	★	REB1 SGDID:S000000253, Chr II from 33681...	YBR049C	92 kDa		16	8	4
22	☑	☆	SEA4 SGDID:S000000200, Chr II from 21293...	YBL104C	118 kDa		0	5	
23	☑	☆	CDC13 SGDID:S000002379, Chr IV from 650...	YDL220C	105 kDa		0	5	
24	☑	☆	EAF1 SGDID:S000002767, Chr IV from 1194...	YDR359C	113 kDa			3	
25	☑	☆	KAP123 SGDID:S000000912, Chr V from 382...	YER110C	123 kDa			3	

For whole list see file 160330_Act_bc_pep_PD_04_05_06.sf3

5. samples 1, 2, 3

Probability Legend:

- over 95%
- 80% to 94%
- 50% to 79%
- 20% to 49%
- 0% to 19%

MS/MS View:

#	Visible?	Starred?	Identified Proteins (53/64)	Accession Number	Molecular Weight	Protein Grouping Ambiguity	200 kDa s01 (F081531)	200 kDa s02 (F081539)	200 kDa s03 (F081519)
1	☑		MYO3 SGDID:S000001612, Chr XI from 2001...	YKL129C	142 kDa	★	13	14	0
2	☑		MYO5 SGDID:S000004715, Chr XIII from 48...	YMR109W	137 kDa	★	38	35	0
3	☑		EDE1 SGDID:S000000143, Chr II from 13204...	YBL047C	151 kDa		15	20	1
4	☑		DNF2 SGDID:S000002500, Chr IV from 6312...	YDR093W	183 kDa	★	15	25	3
5	☑		DNF1 SGDID:S000000968, Chr V from 51274...	YER166W	178 kDa	★	13	23	6
6	☑		PMA1 SGDID:S000002976, Chr VII from 482...	YGL008C	100 kDa		36	39	12
7	☑		PAN1 SGDID:S000001445, Chr IX from 3699...	YIR006C	160 kDa		180	182	18
8	☑		CHC1 SGDID:S000003174, Chr VII from 107...	YGL206C	187 kDa		131	160	19
9	☑		MYO2 SGDID:S000005853, Chr XV from 925...	YOR326W	181 kDa	★	50	52	23
10	☑		YFR016C SGDID:S000001912, Chr VI from 1...	YFR016C	138 kDa		50	0	0
11	☑		RRP5 SGDID:S000004842, Chr XIII from 731...	YMR229C	193 kDa		16	8	1
12	☑		ACT1 SGDID:S000001855, Chr VI from 5437...	YFL039C	42 kDa		2	3	
13	☑		CRN1 SGDID:S000004421, Chr XII from 990...	YLR429W	73 kDa		6	8	
14	☑		MOT1 SGDID:S000006003, Chr XVI from 404...	YPL082C	210 kDa		3	1	

For whole list see file 160330_Act_bc_pep_PD_01_02_03.sf3

Experimental section

Sample preparation

By client, see above

2 uL 10% TFA and 18 uL supernatant (no additional extraction) were transferred into autosampler vials.

Rest of samples stored in freezerT2/ P4

LC-MS Analysis (DDA)/ Orbitrap Velos

Instrument : Easy-nLC 1000 pump with modified New Objective Digital PicoView ion source coupled to an LTQ Orbitrap Fusion, Acclaim PepMap 100 trap-column (75 µm x 2 cm, nanoViper, C18, 3 µm, 100 Å) and PepMap RSLC analytical column (50 µm x 15 cm, C18, 2 µm, all Thermo Fisher Scientific)

Configuration **: two-column set-up** (trap-column and analytical column installed)

Buffer A : 0.1% formic acid in water

Buffer B : 0.1% formic acid in acetonitrile

Methods/ Injection order/ Injection volume – see tables

File Name	Instrument Method	Position	Inj Vol
160401_MPDS_10fmol_1	ddTopT_30min_HCD_IT_Universal_2e5_1ul	1-V1	1
160401_MPDS_1fmol_1	ddTopT_30min_HCD_IT_Universal_2e5_1ul	1-V2	1
**160401_476_1305_s15**	**ddTopT_30min_HCD_IT_Universal_2e5_18ul**	**D7**	**5**
**160401_476_1305_s12**	**ddTopT_30min_HCD_IT_Universal_2e5_18ul**	**D4**	**5**
**160401_476_1305_s09**	**ddTopT_30min_HCD_IT_Universal_2e5_18ul**	**D1**	**5**
**160401_476_1305_s06**	**ddTopT_30min_HCD_IT_Universal_2e5_18ul**	**C6**	**5**
**160401_476_1305_s03**	**ddTopT_30min_HCD_IT_Universal_2e5_18ul**	**C3**	**5**
160401_MPDS_1fmol_2	ddTopT_30min_HCD_IT_Universal_2e5_1ul	1-V2	1
**160401_476_1305_s13**	**ddTopT_30min_HCD_IT_Universal_2e5_18ul**	**D5**	**5**
**160401_476_1305_s10**	**ddTopT_30min_HCD_IT_Universal_2e5_18ul**	**D2**	**5**
**160401_476_1305_s07**	**ddTopT_30min_HCD_IT_Universal_2e5_18ul**	**C7**	**5**
**160401_476_1305_s04**	**ddTopT_30min_HCD_IT_Universal_2e5_18ul**	**C4**	**5**
**160401_476_1305_s01**	**ddTopT_30min_HCD_IT_Universal_2e5_18ul**	**C1**	**5**
160401_MPDS_1fmol_3	ddTopT_30min_HCD_IT_Universal_2e5_1ul	1-V2	1
**160401_476_1305_s14**	**ddTopT_30min_HCD_IT_Universal_2e5_18ul**	**D6**	**5**
**160401_476_1305_s11**	**ddTopT_30min_HCD_IT_Universal_2e5_18ul**	**D3**	**5**
**160401_476_1305_s08**	**ddTopT_30min_HCD_IT_Universal_2e5_18ul**	**C8**	**5**
**160401_476_1305_s05**	**ddTopT_30min_HCD_IT_Universal_2e5_18ul**	**C5**	**5**
**160401_476_1305_s02**	**ddTopT_30min_HCD_IT_Universal_2e5_18ul**	**C2**	**5**
160401_MPDS_1fmol_4	ddTopT_30min_HCD_IT_Universal_2e5_1ul	1-V2	1
160401_MPDS_1fmol_5	ddTopT_30min_HCD_IT_Universal_2e5_1ul	1-V2	1

Mascot search parameter

Database : SGD_yeast orf_trans_all, Contination Contaminants contaminants_cRAPMaxQFMI__150507

Taxonomy : all

Enzyme : Trypsin/P

Fixed modifications : Carbamidomethyl (C)

Variable modifications : Acetyl (Protein N-term), Oxidation (M), Phospho (ST)

Peptide mass tolerance : ± 10 ppm

Fragment mass tolerance : ± 0.6 Da

Max. missed cleavages : 3

Scaffold

File name	Mascot runs
160330_Act_bc_pep_PD_13_14_15.sf3	160401_476_1305_s13.raw (F081523)
	160401_476_1305_s14.raw (F081535)
	160401_476_1305_s15.raw (F081511)
160330_Act_bc_pep_PD_10_11_12.sf3	160401_476_1305_s10.raw (F081524)
	160401_476_1305_s11.raw (F081536)
	160401_476_1305_s12.raw (F081514)
160330_Act_bc_pep_PD_07_08_09.sf3	160401_476_1305_s07.raw (F081525)
	160401_476_1305_s08.raw (F081537)
	160401_476_1305_s09.raw (F081516)
160330_Act_bc_pep_PD_04_05_06.sf3	160401_476_1305_s04.raw (F081530)
	160401_476_1305_s05.raw (F081538)
	160401_476_1305_s06.raw (F081517)
160330_Act_bc_pep_PD_01_02_03.sf3	160401_476_1305_s01.raw (F081531)
	160401_476_1305_s02.raw (F081539)
	160401_476_1305_s03.raw (F081519)
