# Supplementary figures and images for: TORC2 inhibition triggers yeast chromosome fragmentation through misregulated Base Excision Repair of clustered oxidation events
Source: Nat Commun. 2024 Nov 15;15:9908. doi: 10.1038/s41467-024-54142-z (PMC11568337; doi:10.1038/s41467-024-54142-z)

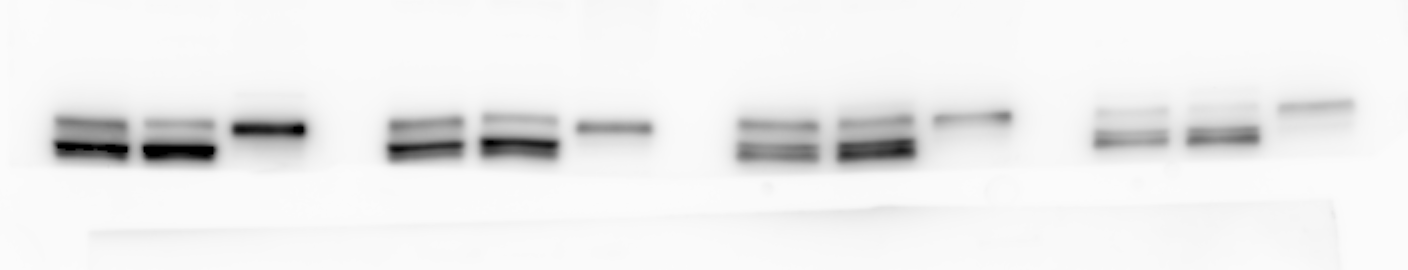

Supplement: Supplementary file 6 — Source Data [file 41467_2024_54142_MOESM6_ESM.zip › uncrop and quant Fig7d/201223_Kenji_Western_arp5_Orc2/chr1_Orc2_2m_crop.tif]

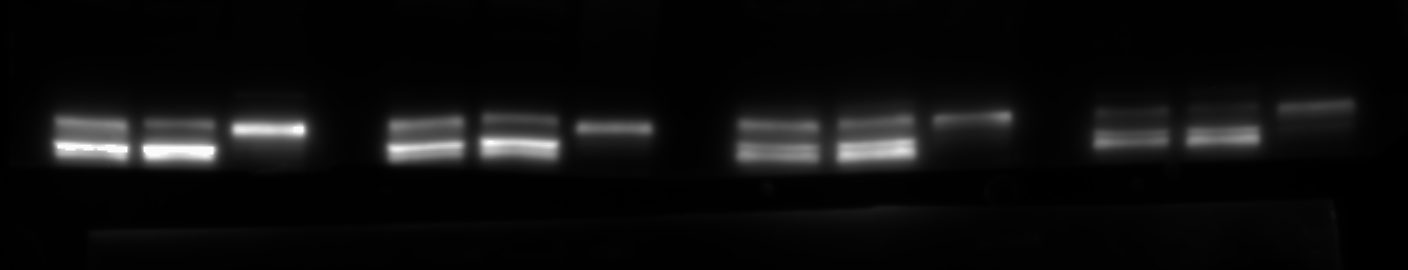

Supplement: Supplementary file 6 — Source Data [file 41467_2024_54142_MOESM6_ESM.zip › uncrop and quant Fig7d/201223_Kenji_Western_arp5_Orc2/chr1_Orc2_2m_crop_inverted.tif]

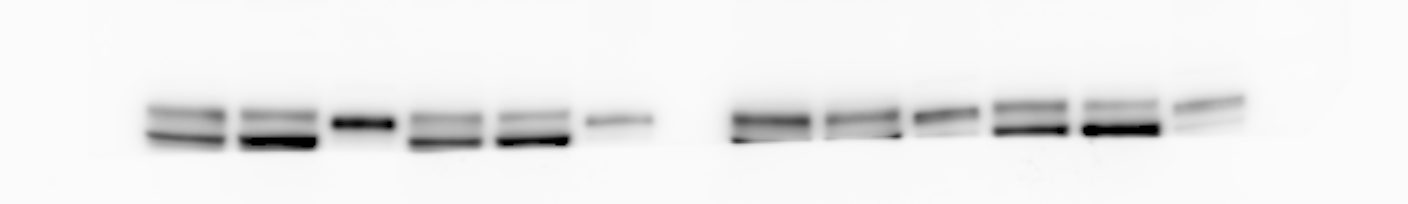

Supplement: Supplementary file 6 — Source Data [file 41467_2024_54142_MOESM6_ESM.zip › uncrop and quant Fig7d/201223_Kenji_Western_arp5_Orc2/chr2_Orc2_2m_crop.tif]

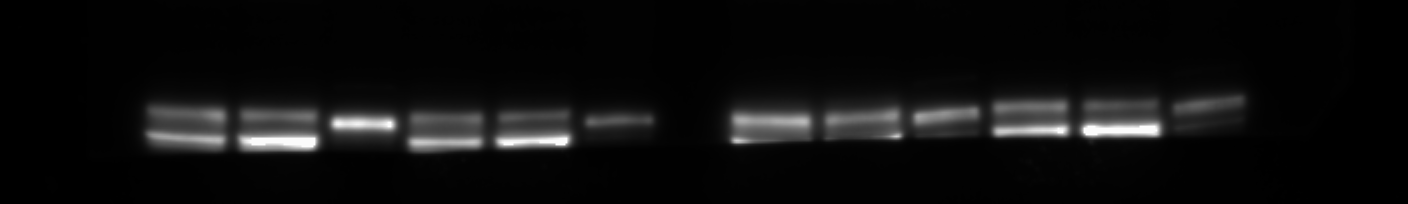

Supplement: Supplementary file 6 — Source Data [file 41467_2024_54142_MOESM6_ESM.zip › uncrop and quant Fig7d/201223_Kenji_Western_arp5_Orc2/chr2_Orc2_2m_crop_inverted.tif]

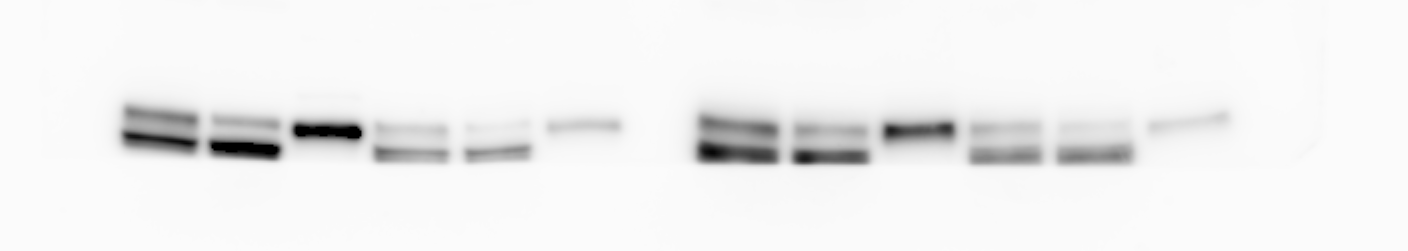

Supplement: Supplementary file 6 — Source Data [file 41467_2024_54142_MOESM6_ESM.zip › uncrop and quant Fig7d/201223_Kenji_Western_arp5_Orc2/chr3_Orc2_2m_crop.tif]

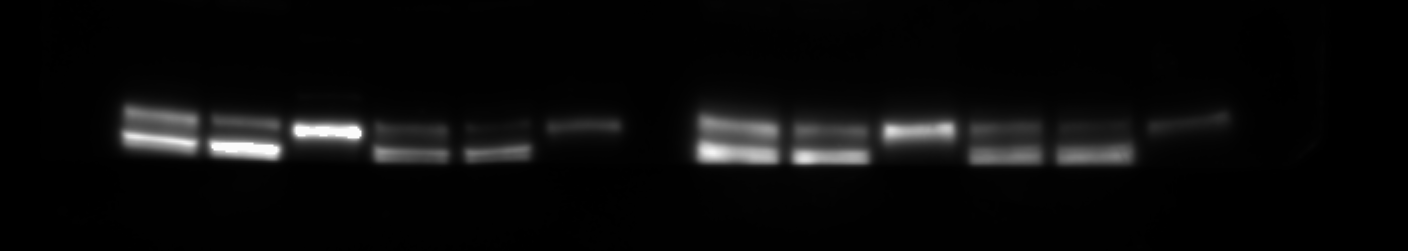

Supplement: Supplementary file 6 — Source Data [file 41467_2024_54142_MOESM6_ESM.zip › uncrop and quant Fig7d/201223_Kenji_Western_arp5_Orc2/chr3_Orc2_2m_crop_inverted.tif]

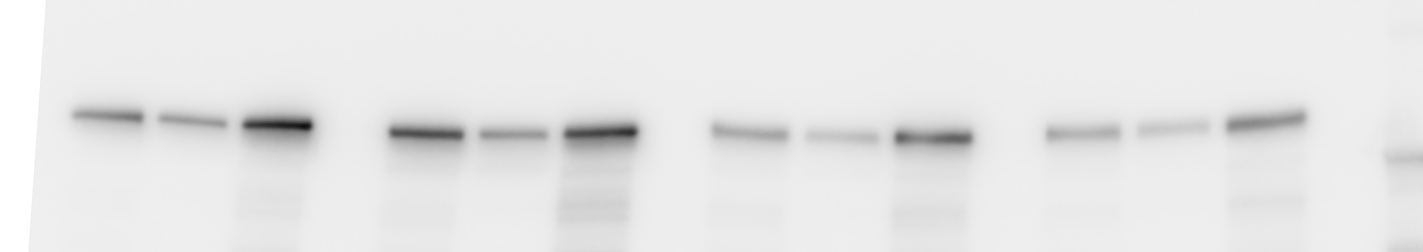

Supplement: Supplementary file 6 — Source Data [file 41467_2024_54142_MOESM6_ESM.zip › uncrop and quant Fig7d/2020.10.16_chr frac_tiffs/a-arp8_APN-chr_2s_crop.tif]

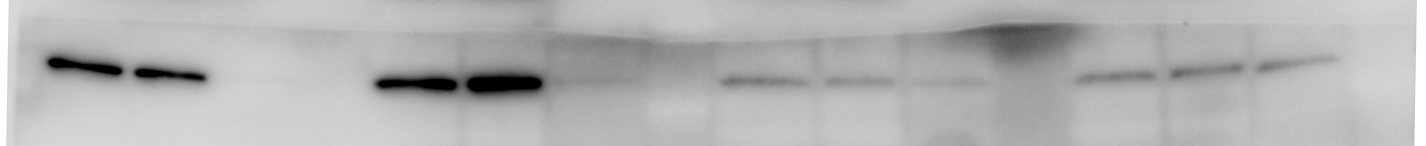

Supplement: Supplementary file 6 — Source Data [file 41467_2024_54142_MOESM6_ESM.zip › uncrop and quant Fig7d/2020.10.16_chr frac_tiffs/actin_2m_crop.tif]

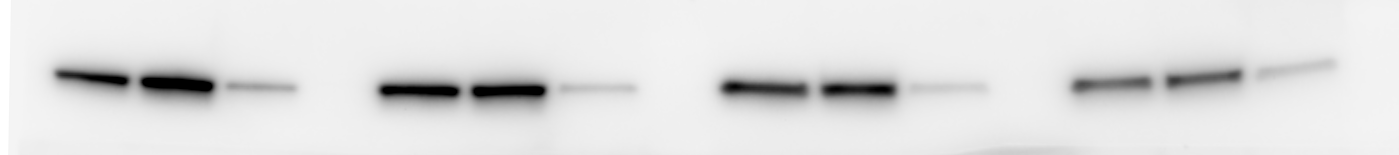

Supplement: Supplementary file 6 — Source Data [file 41467_2024_54142_MOESM6_ESM.zip › uncrop and quant Fig7d/2020.10.16_chr frac_tiffs/APN1_2m_crop.tif]

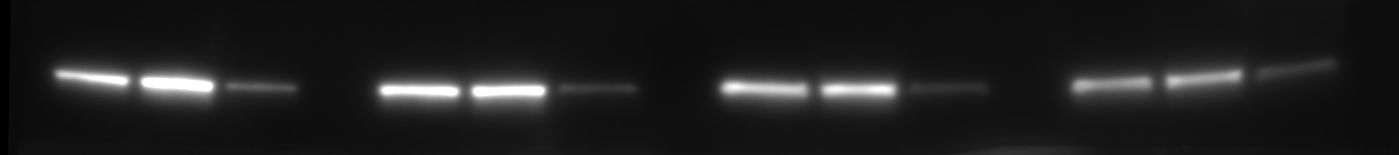

Supplement: Supplementary file 6 — Source Data [file 41467_2024_54142_MOESM6_ESM.zip › uncrop and quant Fig7d/2020.10.16_chr frac_tiffs/APN1_2m_crop_inverted.tif]

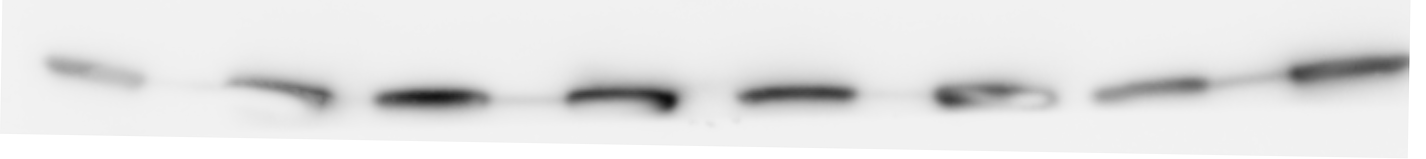

Supplement: Supplementary file 6 — Source Data [file 41467_2024_54142_MOESM6_ESM.zip › uncrop and quant Fig7d/2020.10.16_chr frac_tiffs/H4_0.8_crop.tif]

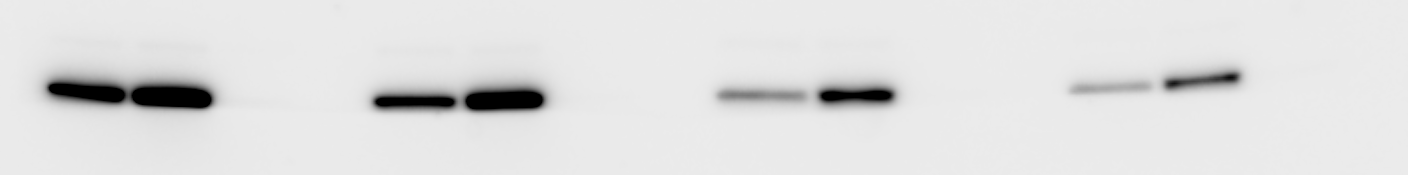

Supplement: Supplementary file 6 — Source Data [file 41467_2024_54142_MOESM6_ESM.zip › uncrop and quant Fig7d/2020.10.16_chr frac_tiffs/tub_40s_crop.tif]

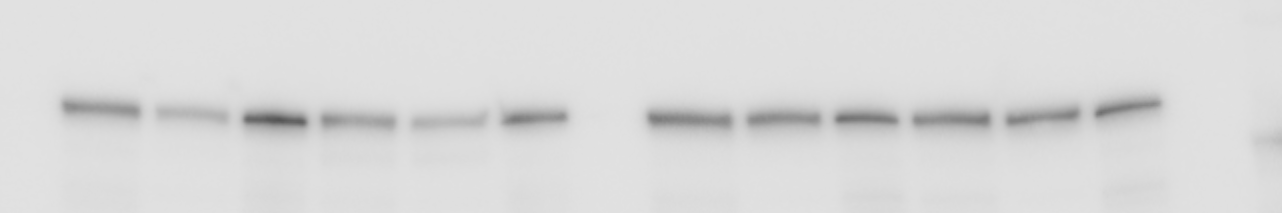

Supplement: Supplementary file 6 — Source Data [file 41467_2024_54142_MOESM6_ESM.zip › uncrop and quant Fig7d/2020.10.22_chr frac_tiffs/a-arp8_OGG-chr_1s_crop.tif]

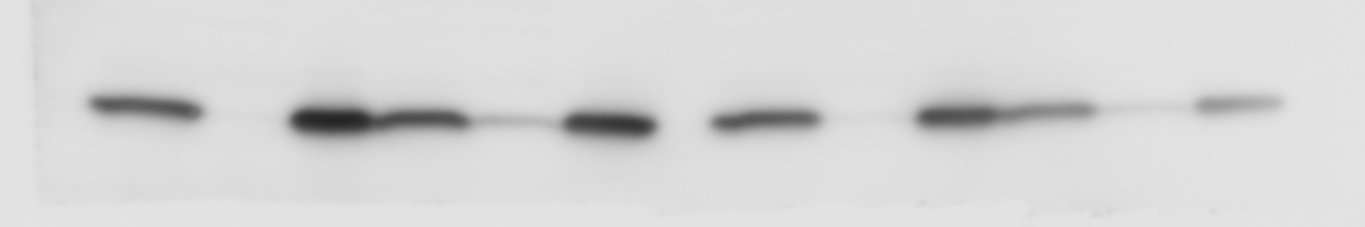

Supplement: Supplementary file 6 — Source Data [file 41467_2024_54142_MOESM6_ESM.zip › uncrop and quant Fig7d/2020.10.22_chr frac_tiffs/Chr_H4_1s_crop.tif]

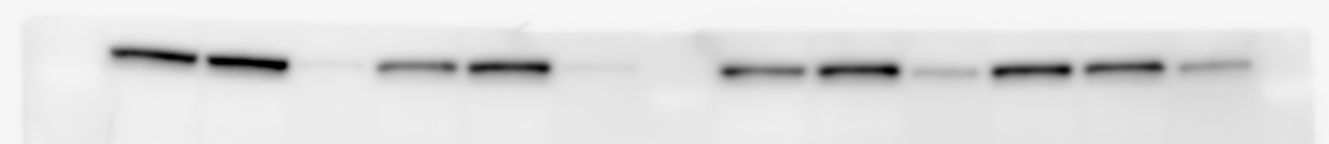

Supplement: Supplementary file 6 — Source Data [file 41467_2024_54142_MOESM6_ESM.zip › uncrop and quant Fig7d/2020.10.22_chr frac_tiffs/chr_m-act_2m 2020.11.06_crop.tif]

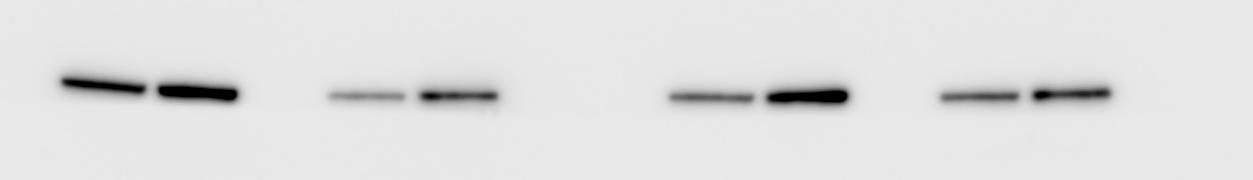

Supplement: Supplementary file 6 — Source Data [file 41467_2024_54142_MOESM6_ESM.zip › uncrop and quant Fig7d/2020.10.22_chr frac_tiffs/chr_tub_30s 2020.11.06_crop.tif]

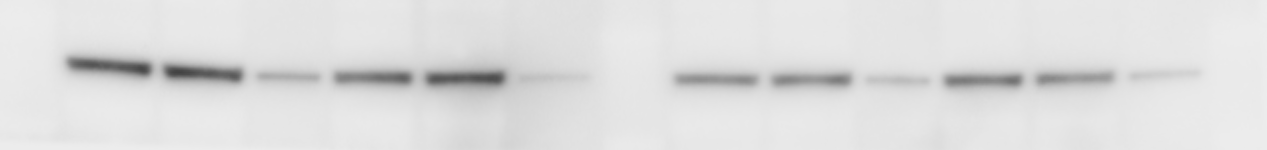

Supplement: Supplementary file 6 — Source Data [file 41467_2024_54142_MOESM6_ESM.zip › uncrop and quant Fig7d/2020.10.22_chr frac_tiffs/OGG1-myc_1m 2020.11.05_crop.tif]

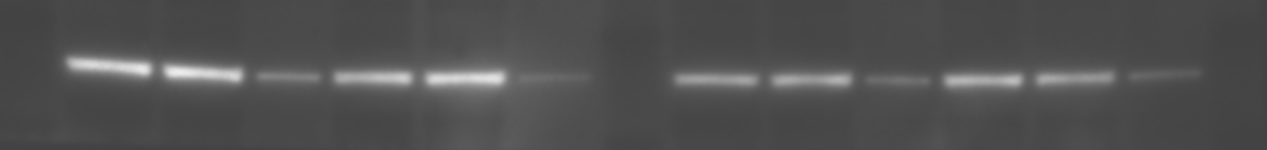

Supplement: Supplementary file 6 — Source Data [file 41467_2024_54142_MOESM6_ESM.zip › uncrop and quant Fig7d/2020.10.22_chr frac_tiffs/OGG1-myc_1m 2020.11.05_crop_inverted.tif]

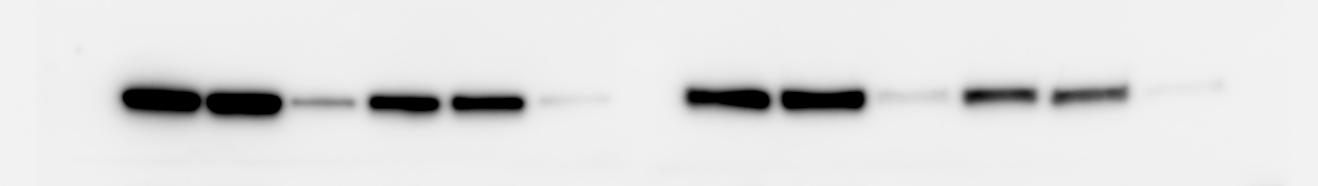

Supplement: Supplementary file 6 — Source Data [file 41467_2024_54142_MOESM6_ESM.zip › uncrop and quant Fig7d/2020.11.20 chr frac_tiffs/Apn1_chr_4m_crop.tif]

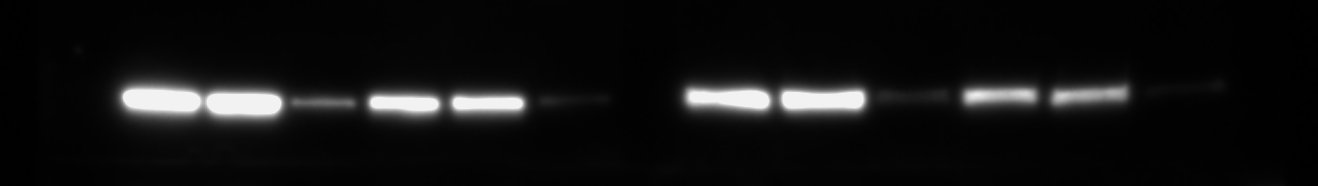

Supplement: Supplementary file 6 — Source Data [file 41467_2024_54142_MOESM6_ESM.zip › uncrop and quant Fig7d/2020.11.20 chr frac_tiffs/Apn1_chr_4m_crop_inverted.tif]

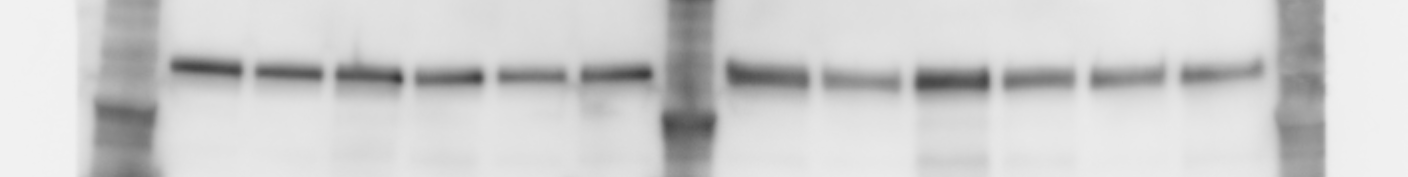

Supplement: Supplementary file 6 — Source Data [file 41467_2024_54142_MOESM6_ESM.zip › uncrop and quant Fig7d/2020.11.20 chr frac_tiffs/chr arp5_3s_crop.tif]

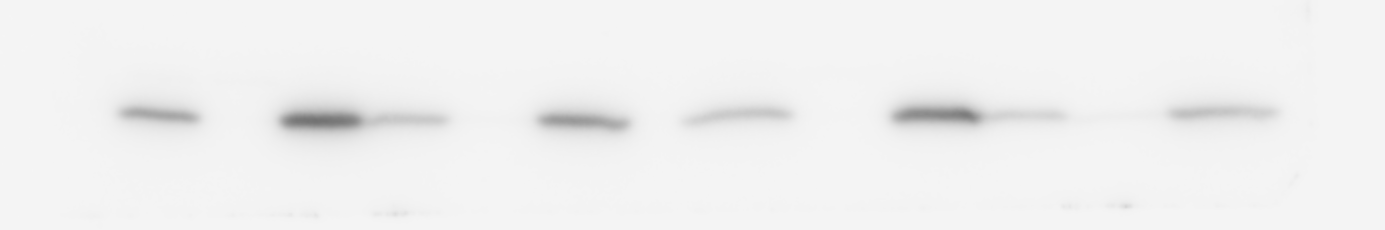

Supplement: Supplementary file 6 — Source Data [file 41467_2024_54142_MOESM6_ESM.zip › uncrop and quant Fig7d/2020.11.20 chr frac_tiffs/chr_set2_H4_2s_crop.tif]

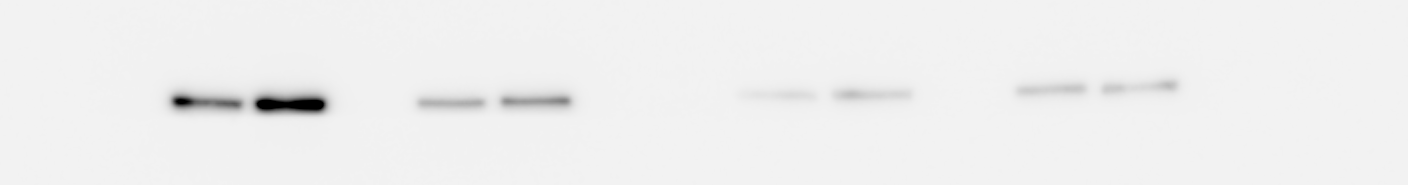

Supplement: Supplementary file 6 — Source Data [file 41467_2024_54142_MOESM6_ESM.zip › uncrop and quant Fig7d/2020.11.20 chr frac_tiffs/set2_tub_1m_crop.tif]

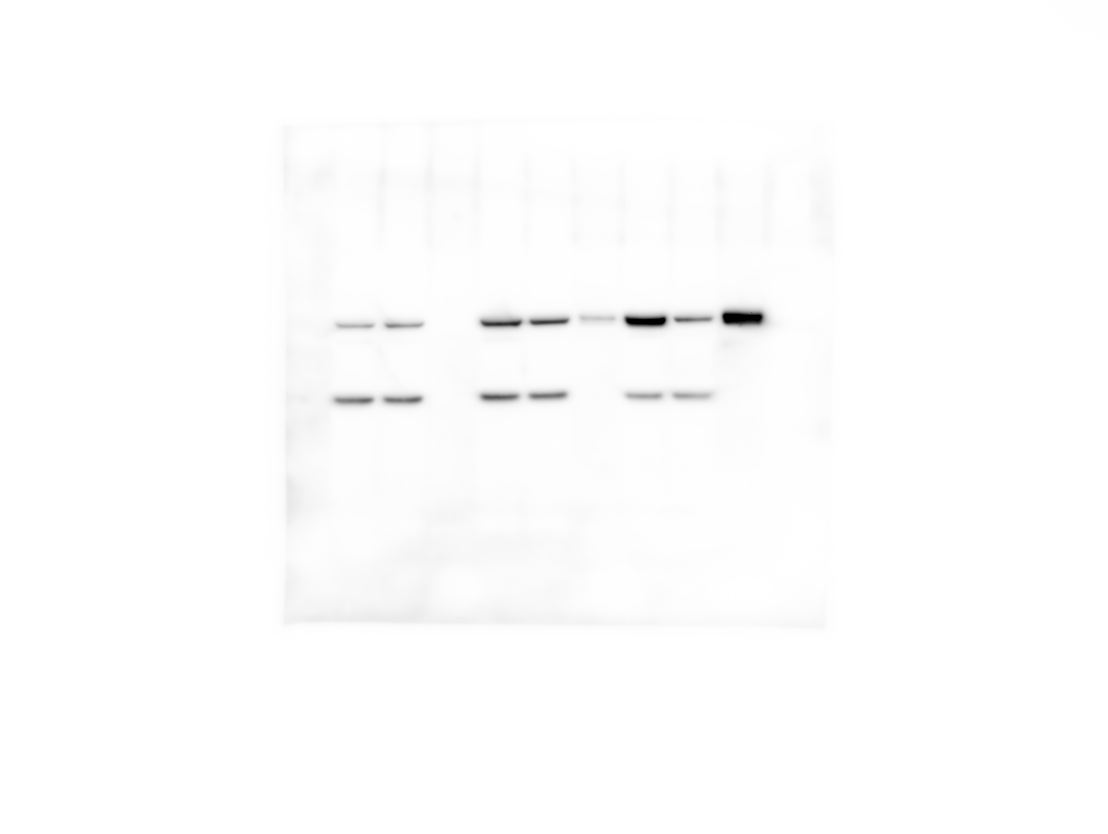

Supplement: Supplementary file 6 — Source Data [file 41467_2024_54142_MOESM6_ESM.zip › uncrop_picts_Fig6b/170307 act 3_full.tif]

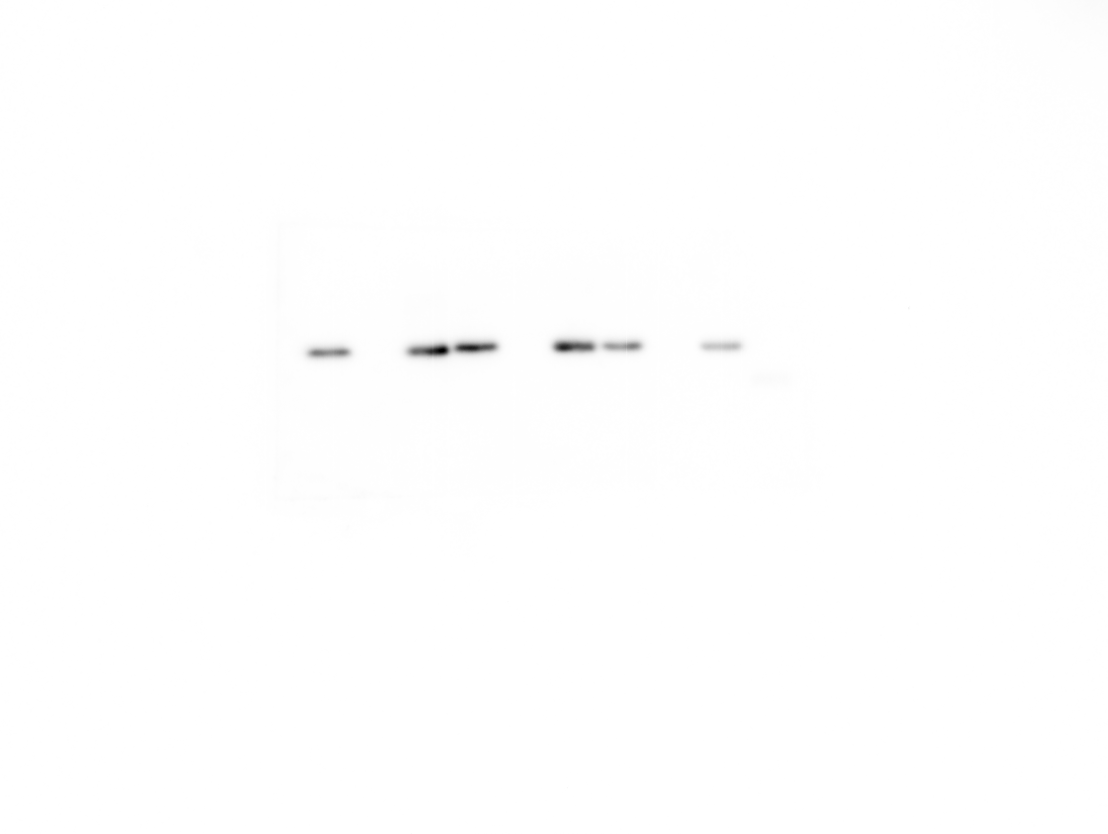

Supplement: Supplementary file 6 — Source Data [file 41467_2024_54142_MOESM6_ESM.zip › uncrop_picts_Fig6b/170307 h4 3_full.tif]

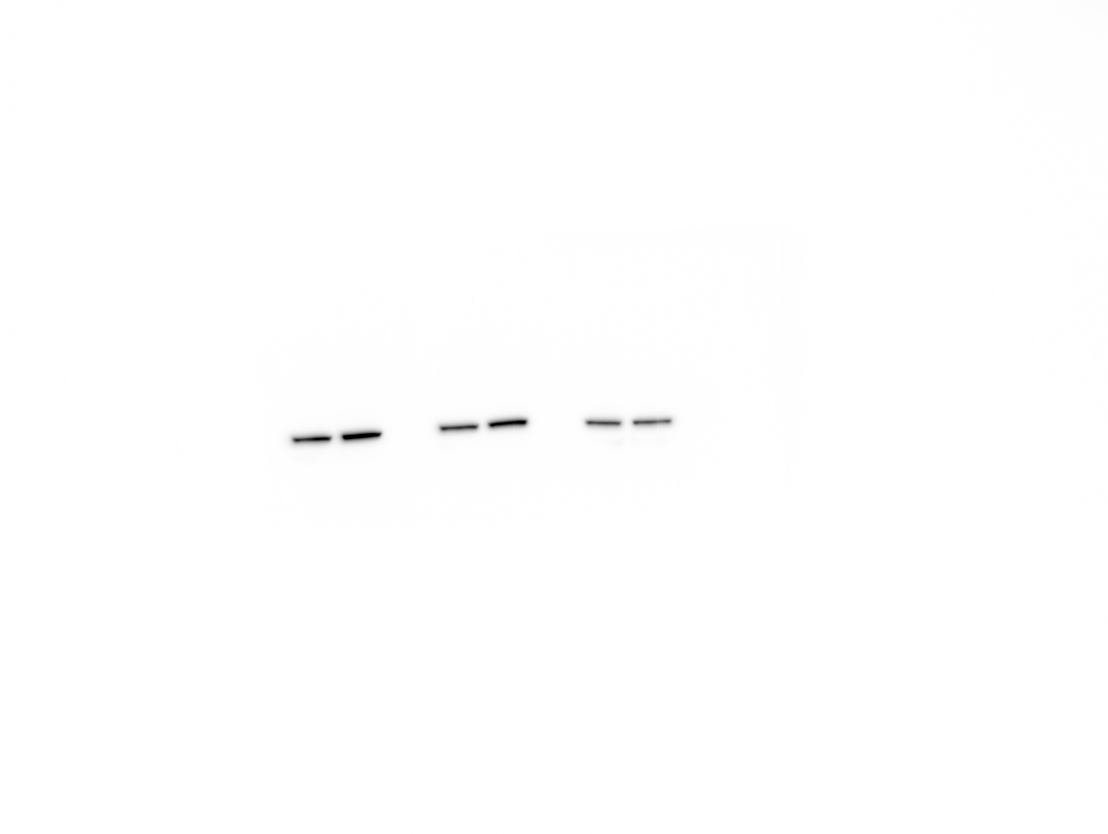

Supplement: Supplementary file 6 — Source Data [file 41467_2024_54142_MOESM6_ESM.zip › uncrop_picts_Fig6b/170307 tub3_full.tif]

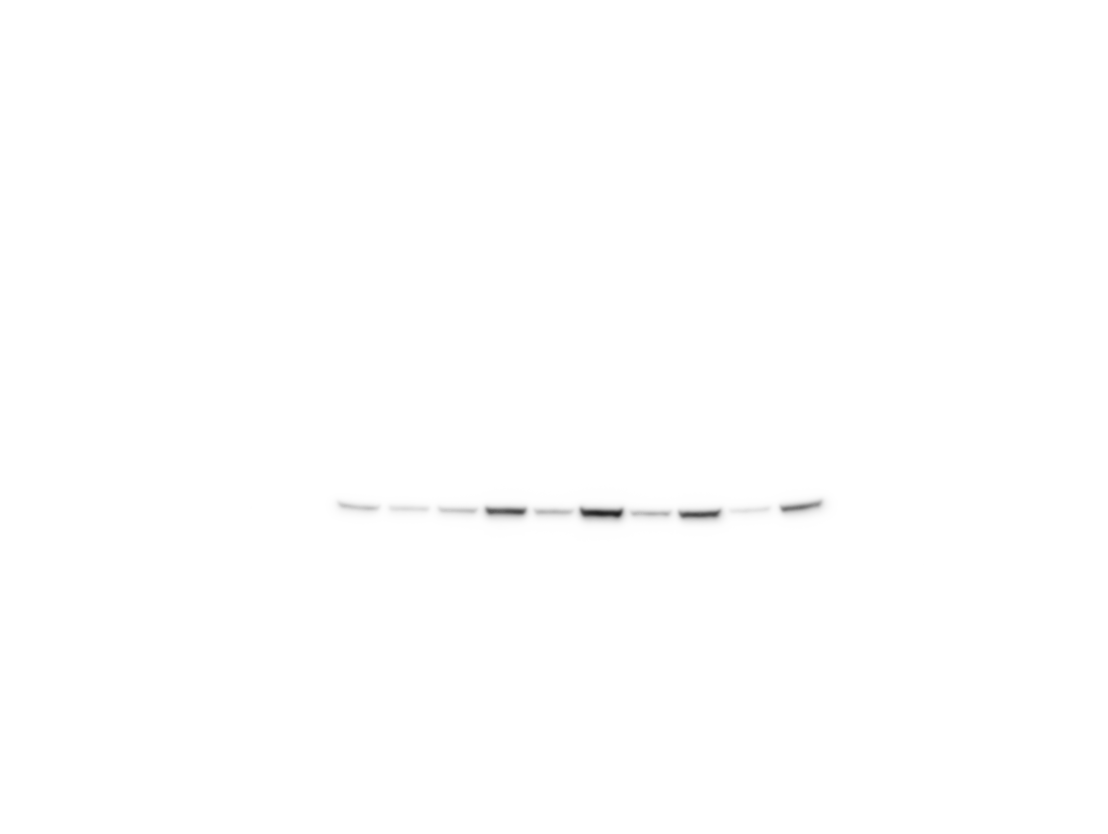

Supplement: Supplementary file 6 — Source Data [file 41467_2024_54142_MOESM6_ESM.zip › uncrop_picts_Fig6c/160805 actin oe total.tif]

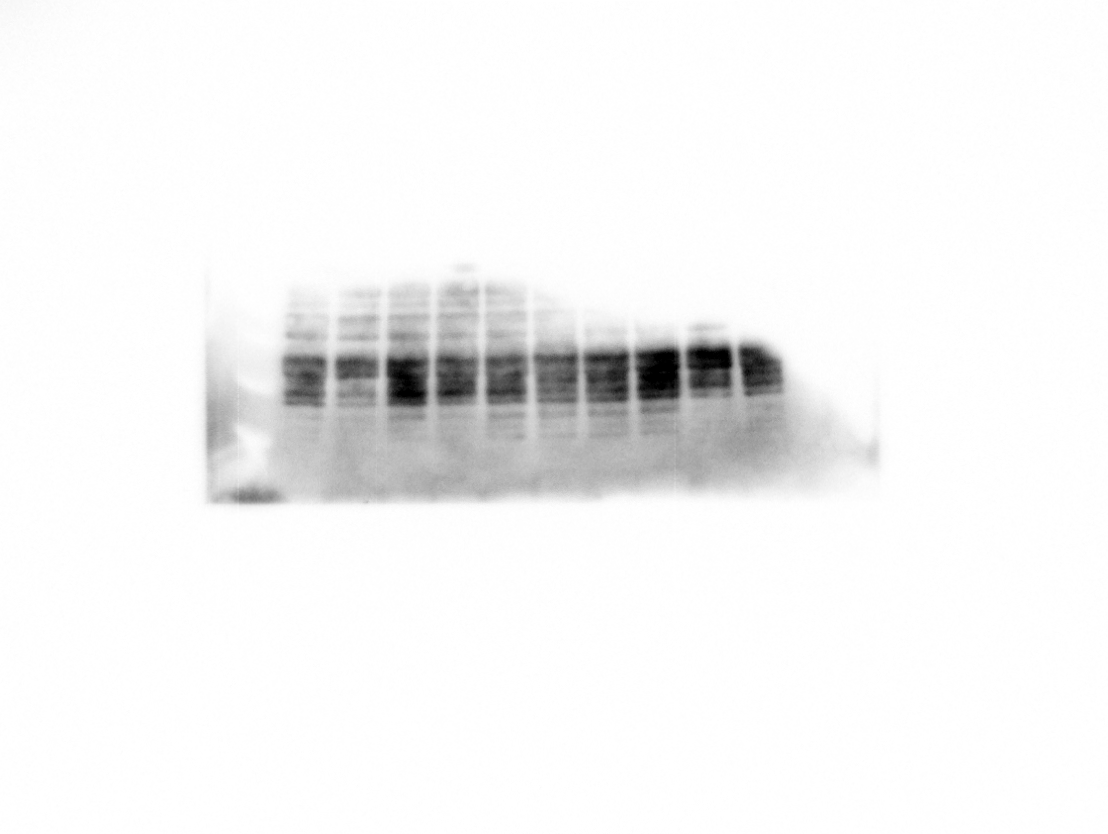

Supplement: Supplementary file 6 — Source Data [file 41467_2024_54142_MOESM6_ESM.zip › uncrop_picts_Fig6c/160805 h3 actin oe.tif]

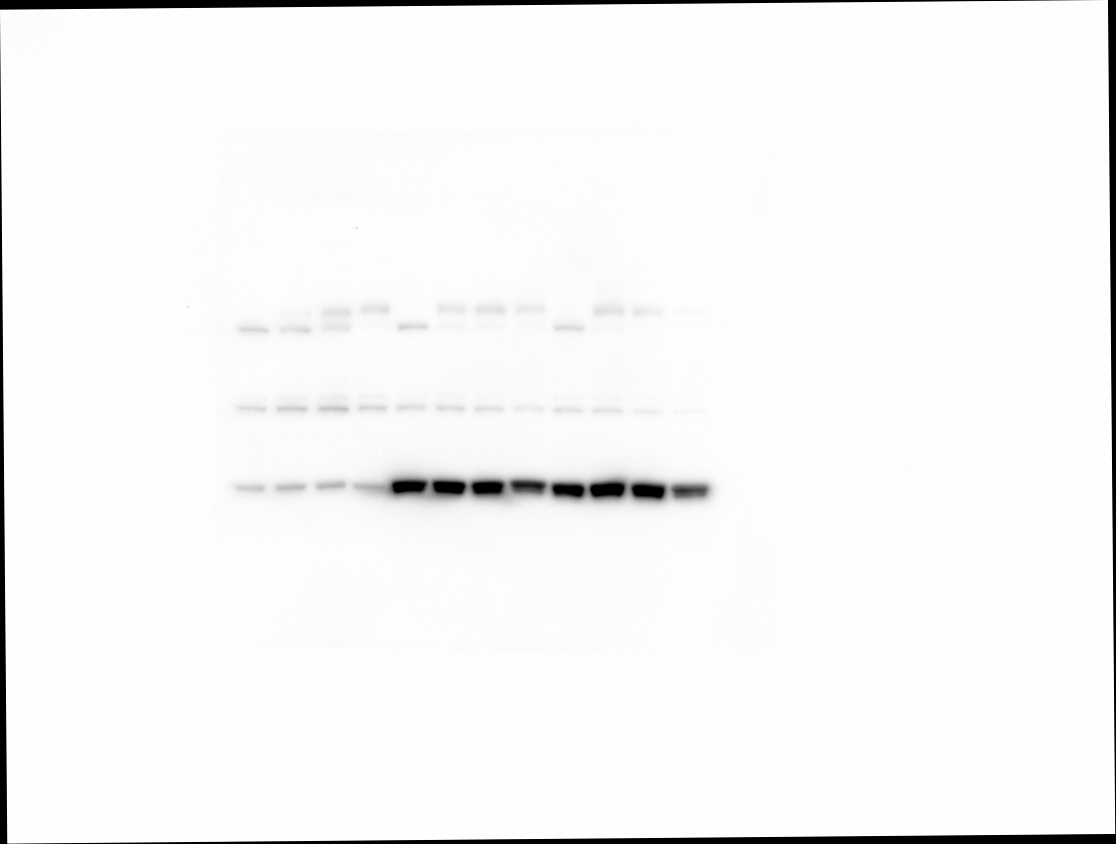

Supplement: Supplementary file 6 — Source Data [file 41467_2024_54142_MOESM6_ESM.zip › uncrop_picts_Fig6d/2016-05-19_actin_set1(v_nesact_nesS14C).tif]

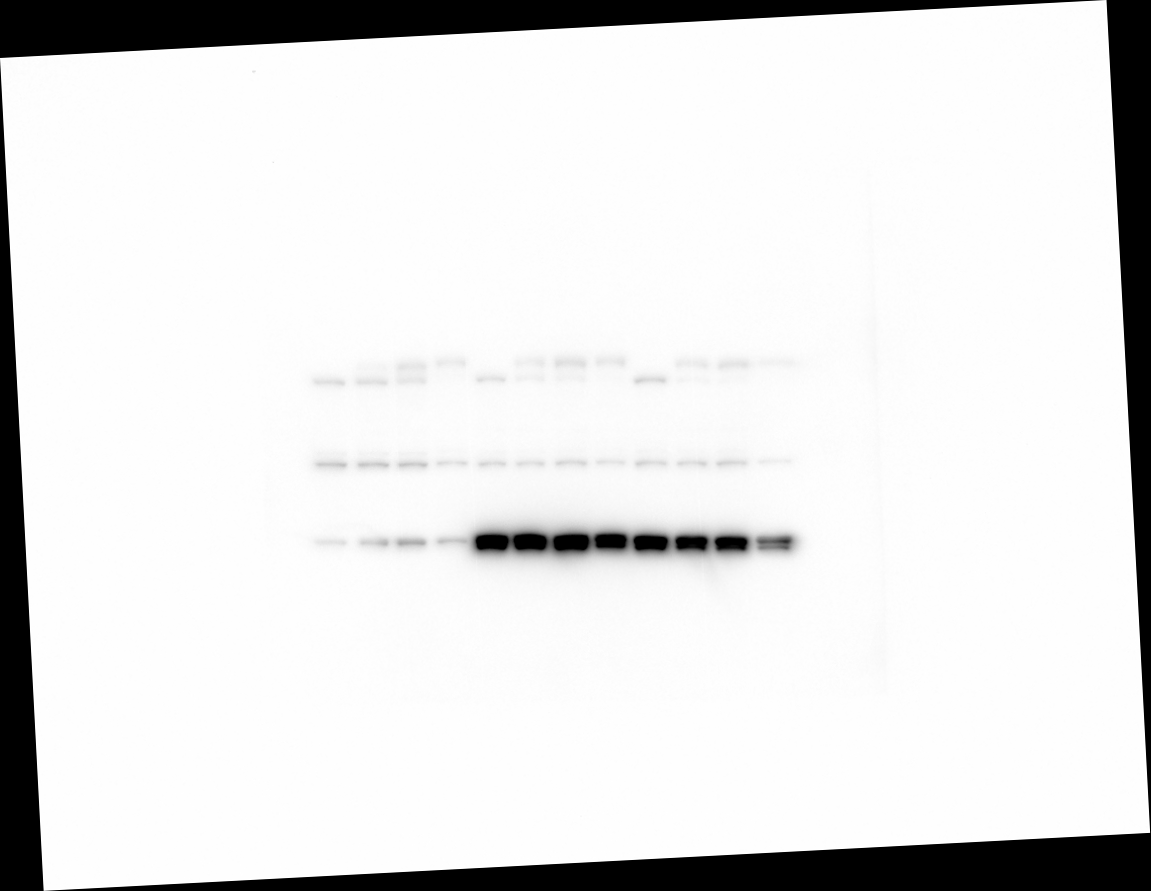

Supplement: Supplementary file 6 — Source Data [file 41467_2024_54142_MOESM6_ESM.zip › uncrop_picts_Fig6d/2016-05-19_actin_set2(v-nesAP_nesact111).tif]

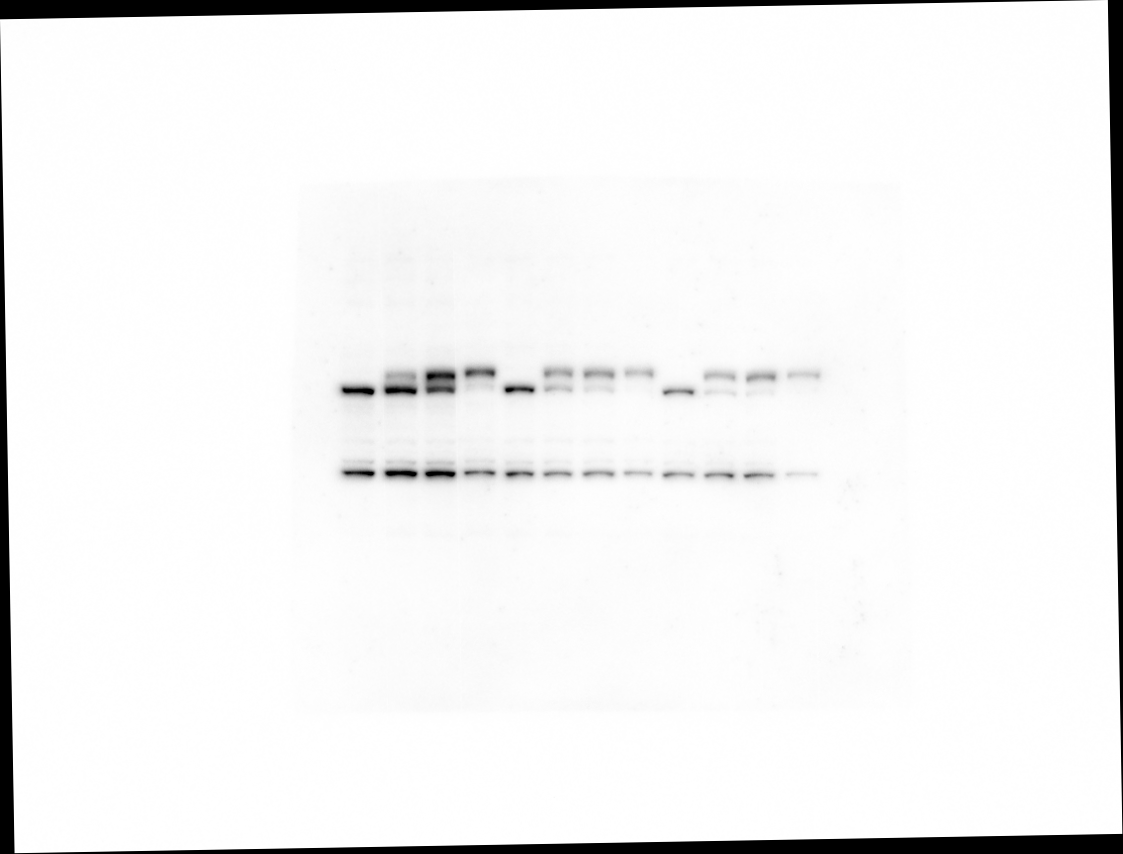

Supplement: Supplementary file 6 — Source Data [file 41467_2024_54142_MOESM6_ESM.zip › uncrop_picts_Fig6d/2016-05-19_rad53-set2(v-nesAP_nesact111).tif]

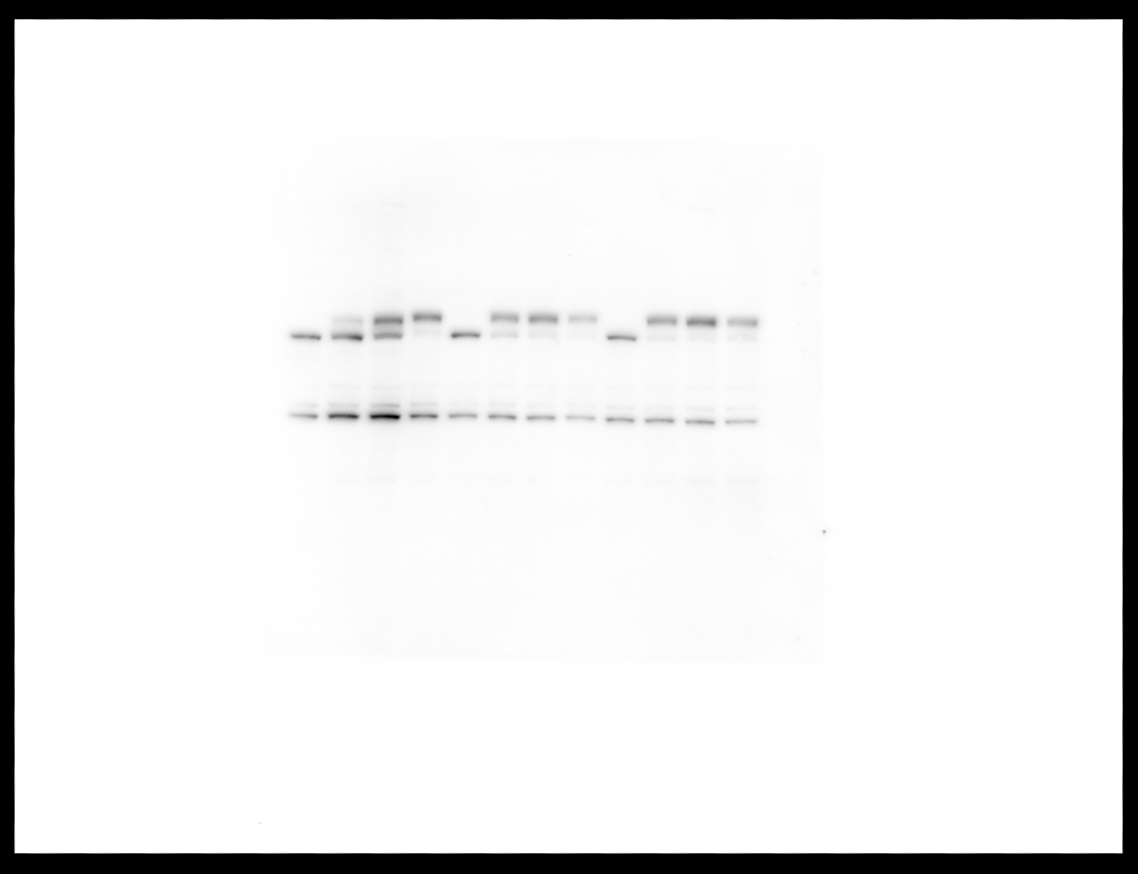

Supplement: Supplementary file 6 — Source Data [file 41467_2024_54142_MOESM6_ESM.zip › uncrop_picts_Fig6d/2016-05-19_rad53_set1(v_nesact_nesS14C).tif]

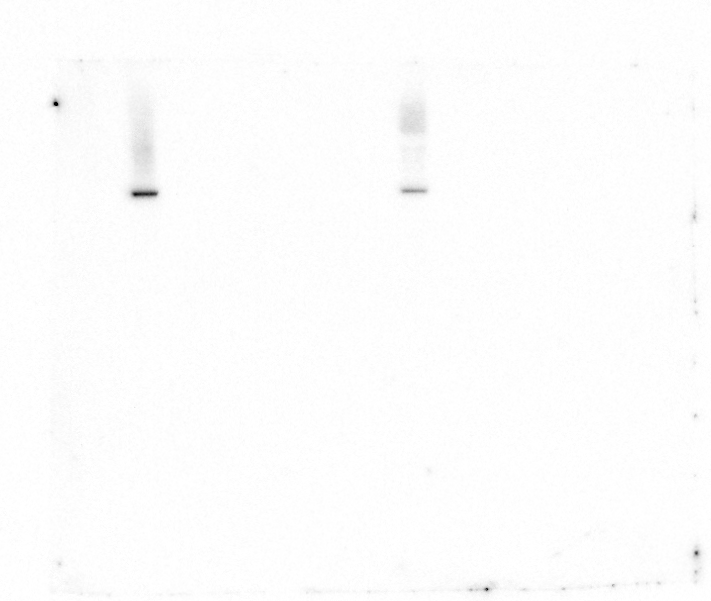

Supplement: Supplementary file 6 — Source Data [file 41467_2024_54142_MOESM6_ESM.zip › uncrop_picts_Fig7a/2016-02_24-mcm2_full.tif]

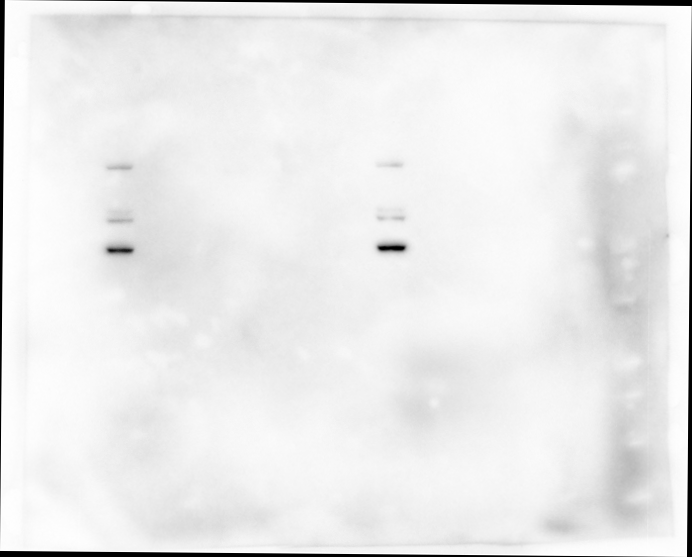

Supplement: Supplementary file 6 — Source Data [file 41467_2024_54142_MOESM6_ESM.zip › uncrop_picts_Fig7a/2016-02_24_anti-tub_2.tif]

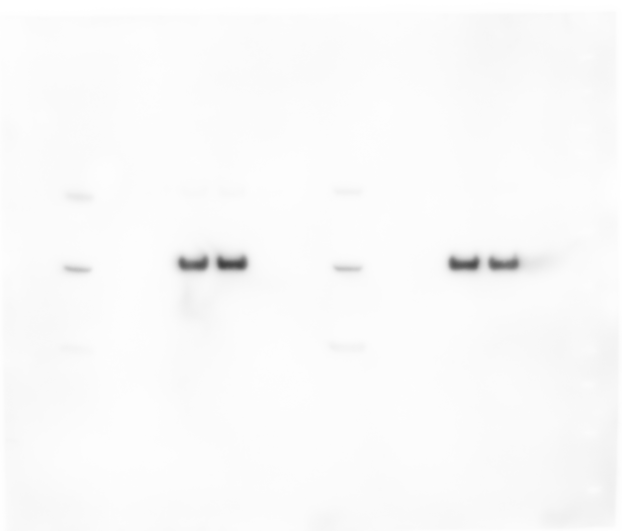

Supplement: Supplementary file 6 — Source Data [file 41467_2024_54142_MOESM6_ESM.zip › uncrop_picts_Fig7a/2016_02_24_anti-actin.tif]

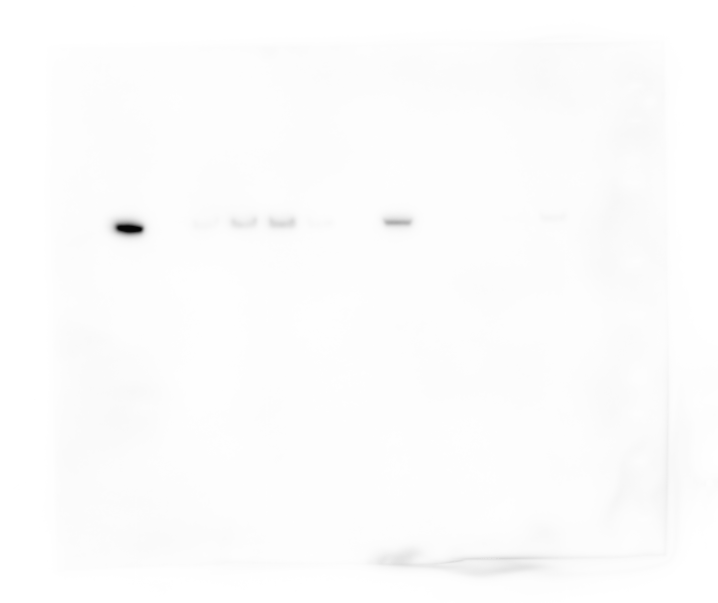

Supplement: Supplementary file 6 — Source Data [file 41467_2024_54142_MOESM6_ESM.zip › uncrop_picts_Fig7a/2016_02_24_anti_myc_full.tif]

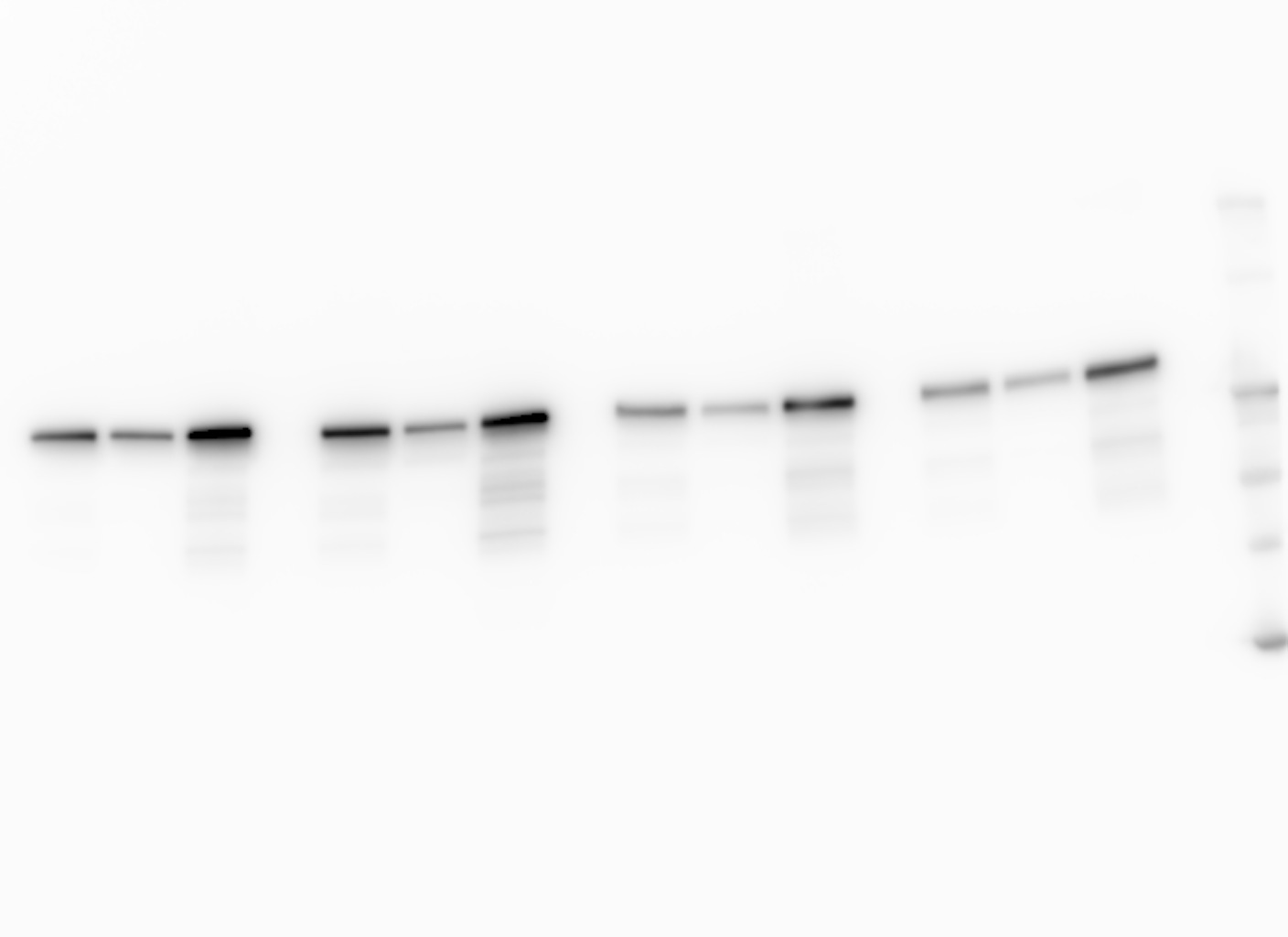

Supplement: Supplementary file 6 — Source Data [file 41467_2024_54142_MOESM6_ESM.zip › uncrop_picts_Fig7c/a-arp5.tif]

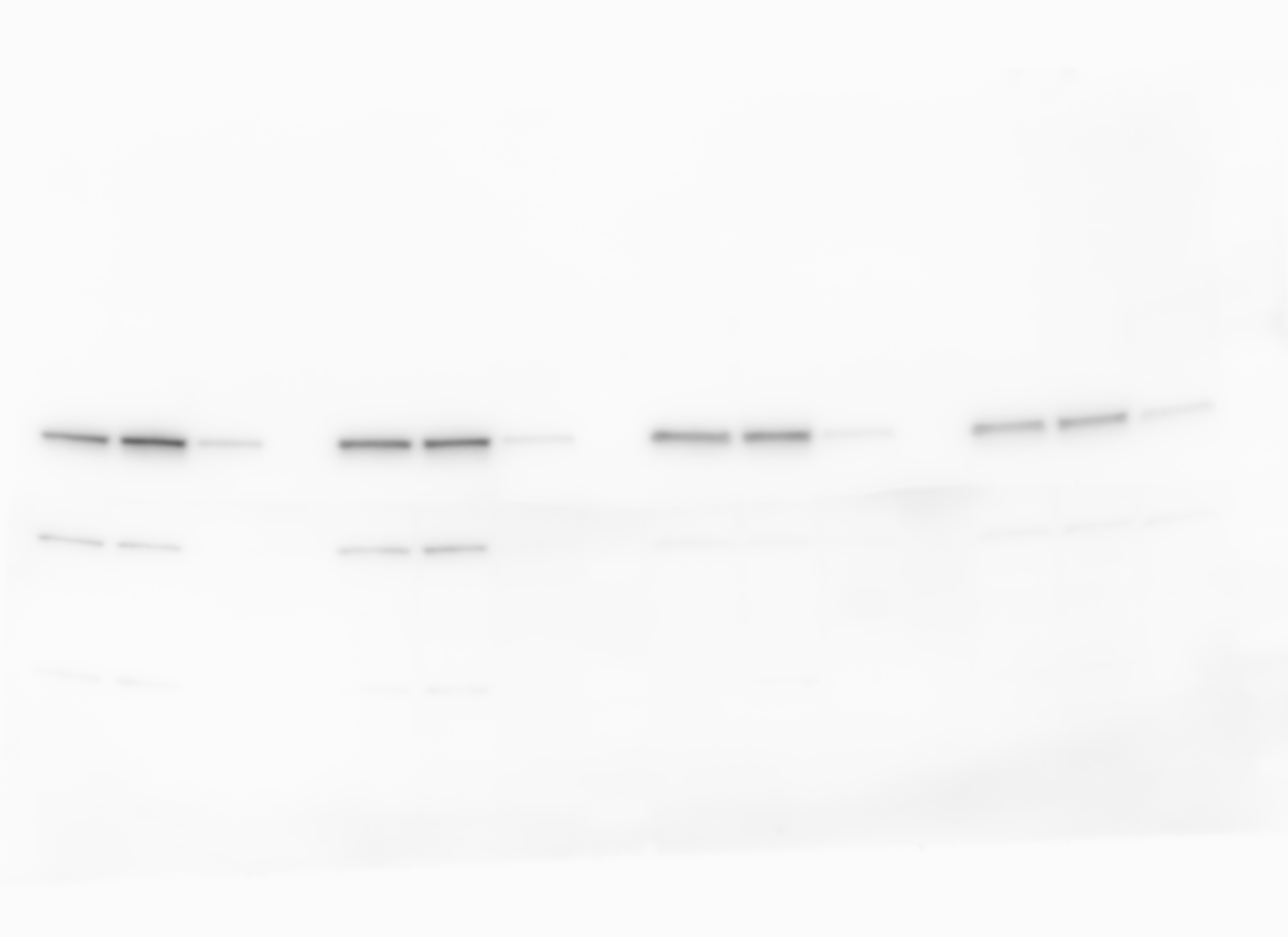

Supplement: Supplementary file 6 — Source Data [file 41467_2024_54142_MOESM6_ESM.zip › uncrop_picts_Fig7c/a_APN1.tif]

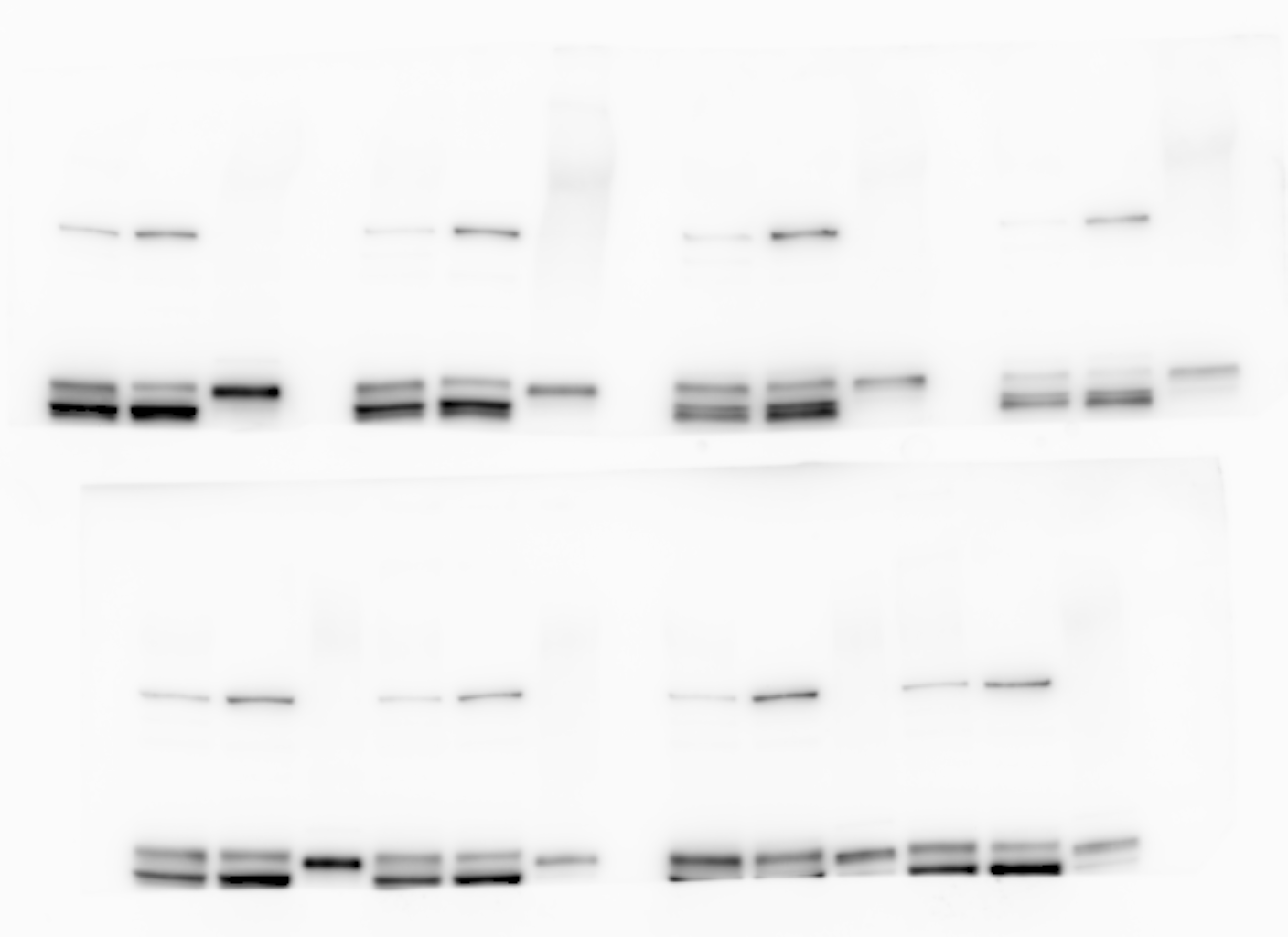

Supplement: Supplementary file 6 — Source Data [file 41467_2024_54142_MOESM6_ESM.zip › uncrop_picts_Fig7c/a_ORC2.tif]

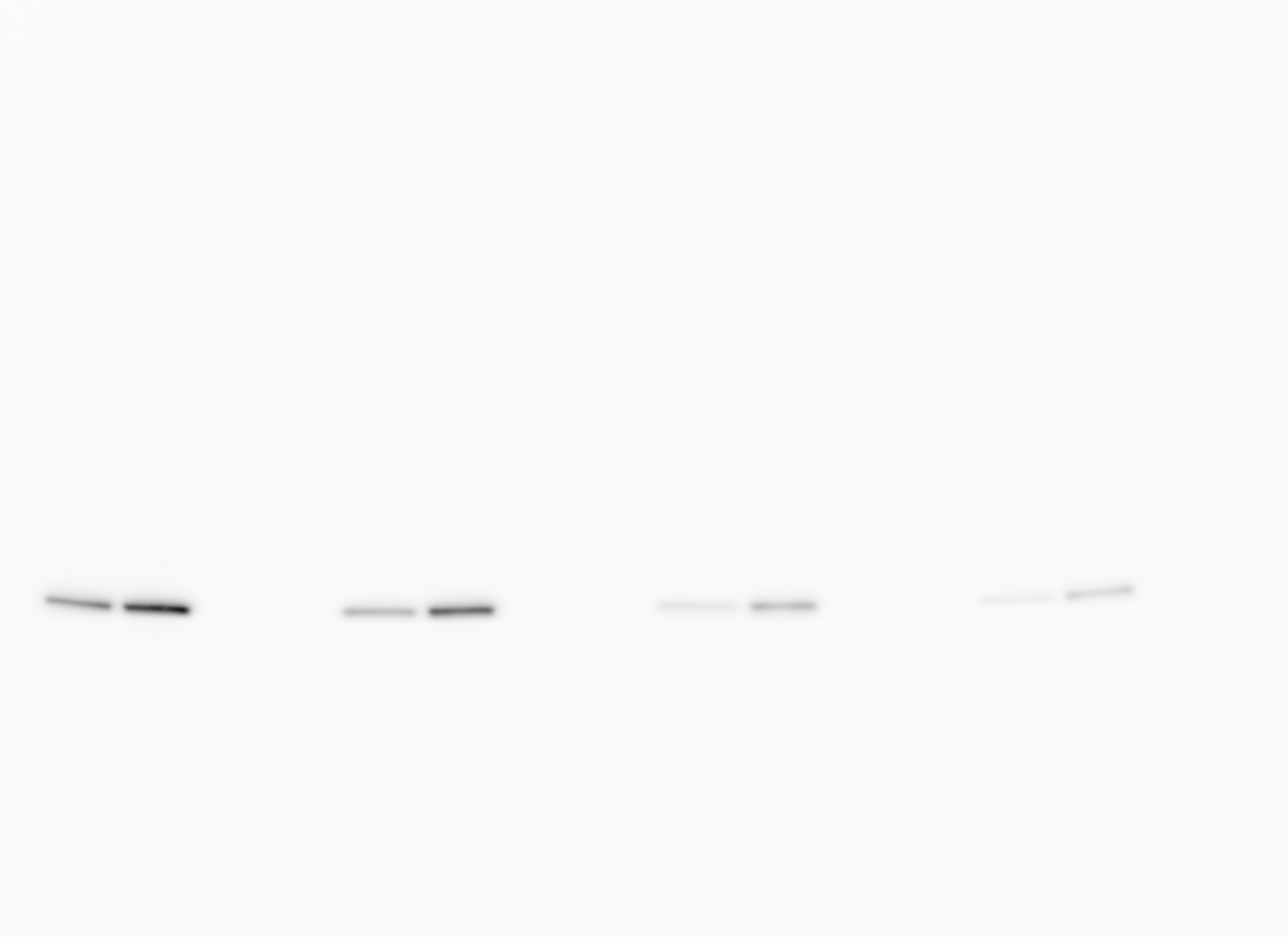

Supplement: Supplementary file 6 — Source Data [file 41467_2024_54142_MOESM6_ESM.zip › uncrop_picts_Fig7c/a_tub.tif]
